# Platelet-rich plasma in the pathologic processes of tendinopathy: a review of basic science studies

**DOI:** 10.3389/fbioe.2023.1187974

**Published:** 2023-07-21

**Authors:** Jialin Lu, Han Li, Ziyu Zhang, Rui Xu, Jincheng Wang, Hui Jin

**Affiliations:** ^1^ Department of Pain, The Second Hospital of Jilin University, Changchun, China; ^2^ Norman Bethune Health Science Center of Jilin University, Changchun, China; ^3^ Department of Endocrinology and Metabolism, Ruijin Hospital, Shanghai Jiao Tong University, Shanghai, China; ^4^ Department of Orthopedics, The Second Hospital of Jilin University, Changchun, China

**Keywords:** platelet-rich plasma, tendinopathy, pathogenesis, healing process, cell proliferation, differentiation

## Abstract

Tendinopathy is a medical condition that includes a spectrum of inflammatory and degenerative tendon changes caused by traumatic or overuse injuries. The pathological mechanism of tendinopathy has not been well defined, and no ideal treatment is currently available. Platelet-rich plasma (PRP) is an autologous whole blood derivative containing a variety of cytokines and other protein components. Various basic studies have found that PRP has the therapeutic potential to promote cell proliferation and differentiation, regulate angiogenesis, increase extracellular matrix synthesis, and modulate inflammation in degenerative tendons. Therefore, PRP has been widely used as a promising therapeutic agent for tendinopathy. However, controversies exist over the optimal treatment regimen and efficacy of PRP for tendinopathy. This review focuses on the specific molecular and cellular mechanisms by which PRP manipulates tendon healing to better understand how PRP affects tendinopathy and explore the reason for the differences in clinical trial outcomes. This article has also pointed out the future direction of basic research and clinical application of PRP in the treatment of tendinopathy, which will play a guiding role in the design of PRP treatment protocols for tendinopathy.

## 1 Introduction

A tendon is a dense connective tissue that connects muscle to bone. It comprises bundles of parallel collagen fibers that can carry tensile loads. The tendon has the function of stabilizing the joint and realizing the joint motion. Therefore, it is usually subjected to heavy mechanical loads that may lead to injury and dysfunction (Millar et al., 2021). Tendinopathy is a common musculoskeletal disorder that is more likely to occur in professional athletes or the middle-aged and elderly population participating in sports. Tendinopathy is characterized by pain, swelling, and dysfunction of the tendon and peritendinous tissue, with the accumulation of lipids, proteoglycans, and calcium in the tendon (Nourissat et al., 2015a). Eventually, tendinopathy will lead to long-term or permanent deficits in the patient’s motor system function. The incidence of tendinopathy has been increasing around the world since the 21st century. About 30% of current clinical consultations for musculoskeletal disorders involve tendinopathy, and about 16.4 million people seek medical intervention each year in orthopedic clinics in the United States alone (James et al., 2008; Macedo et al., 2019). To date, the pathological mechanism of tendinopathy has not been clarified. Research progress in tendinopathy lags behind that in inflammatory arthritis, osteoarthritis, and other motor system diseases. Current hypotheses on tendinopathy pathogenesis describe tendinopathy as a degenerative or failed healing process (Cook and Purdam, 2009; Cook and Purdam, 2021). Degenerative pathology emphasizes apoptosis and matrix degradation of the affected tendon tissue (Cook and Purdam, 2009). Failed healing pathology is characterized by an excessive amount of non-collagenous extracellular matrix, collagen fiber disruption, chronic inflammation, and disordered arrangement of neovascularization within the tendon (Cook and Purdam, 2009). The scar tissue is formed by the deposition of a large number of interlaced or disordered collagen fibers. This will negatively affect the reconstruction of tendon structure and the recovery of tendon biomechanical characteristics, leading to a higher probability of secondary tendon injury (Katzel et al., 2011). Furthermore, scar tissue can cause adhesion and contraction of the tendon, which will significantly impair the inherent function of the tendon and limit the motion range of the joint (Wong et al., 2009).

**SCHEME 1:**
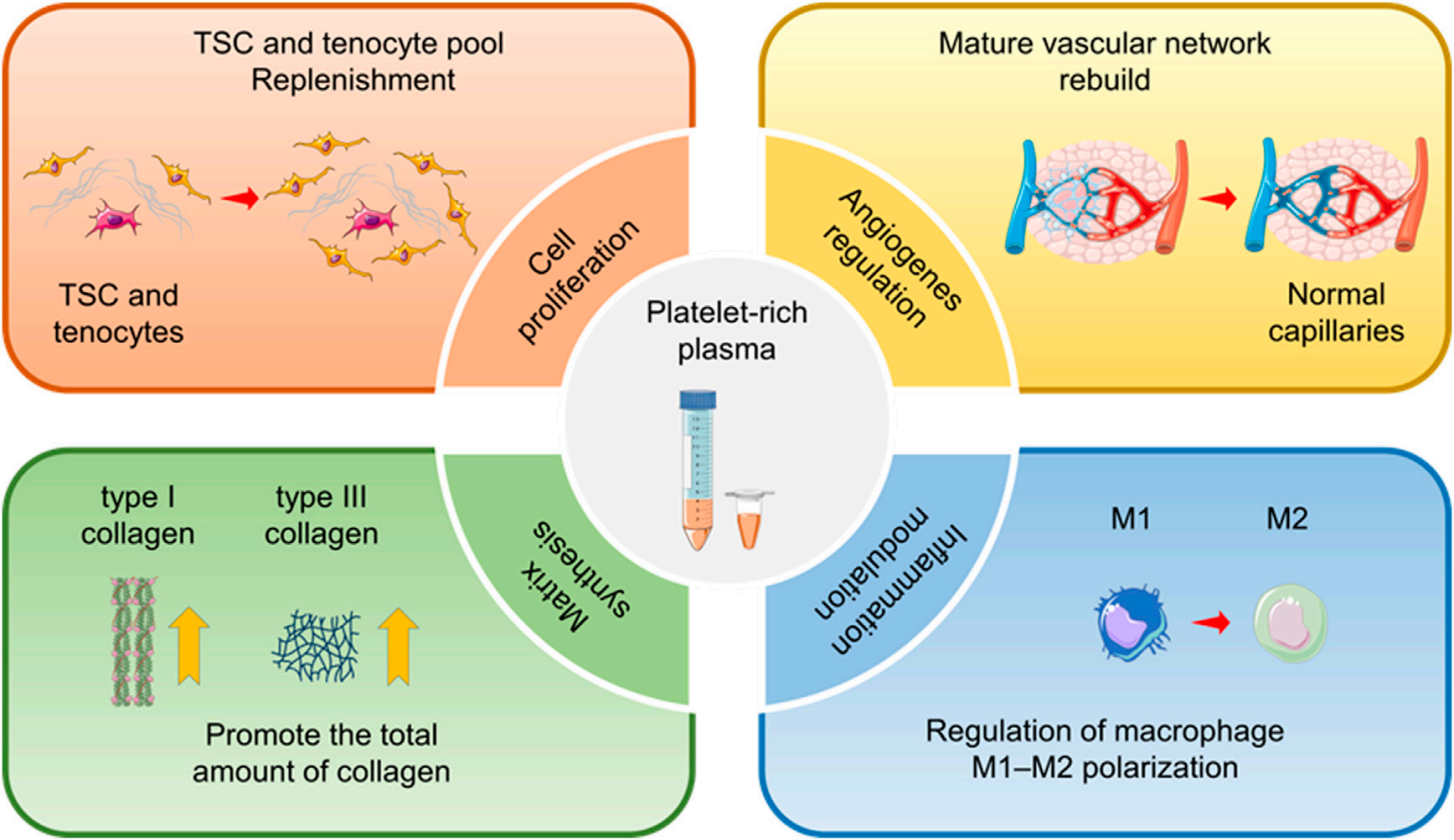
Therapeutic effects of platelet-rich plasma (PRP) for tendinopathy. For chronic tendinopathy, PRP has the therapeutic potential to promote cell proliferation and differentiation, regulate angiogenesis, increase extracellular matrix synthesis, and modulate inflammation. Therefore, PRP has been widely used in the clinical practice of tendinopathy treatment.

Treatment of tendinopathy usually requires clinical intervention. The blood and nutrition supply of tendon is not abundant, and neurotrophin and is always in a high-stress state, which leads to limited self-healing ability. Achilles tendinopathy, patellar tendinopathy, rotator cuff tendinopathy, and lateral elbow tendinopathy are the most common tendinopathy without ideal treatment currently available (Ruan et al., 2021). The clinical treatment strategies of tendinopathy are mainly based on non-surgical treatments, including cold therapy, rest, fixation, non-steroidal anti-inflammatory drug therapy, rehabilitation therapy, and extracorporeal shock wave therapy, to relieve the pain and slow the progress of the degenerative process (Andia et al., 2018). However, most conservative therapies can only be effective in the short term with suboptimal long-term outcomes. Approximately 24%–45.5% of patients eventually require surgical treatment to relieve pain and recover tendon function (Roche and Calder, 2013; Schemitsch et al., 2019). Surgical repair of the tendon can cause complications, such as wound infection, tendon stiffness, adhesions, pain, and swelling. A significant proportion of patients are at risk of re-tearing (Chung et al., 2013; Brockmeyer et al., 2015). Therefore, it is necessary to deeply investigate the pathological process of tendinopathy and the mechanism of tendon healing to update the current treatment therapy. In recent years, platelet-rich plasma (PRP) has emerged as a popular option for the treatment of motor system diseases, including tendinopathy (Zhou and Wang, 2016). The platelet concentration in PRP is usually five times the physiological plasma level (100–300 × 10^9^ cells/L), which can form a local cytokine-rich microenvironment for tendon regeneration (Basdelioglu et al., 2020; Lyu et al., 2021). After activation, platelets in PRP release a variety of growth factors, including platelet-derived growth factor (PDGF), transforming growth factor-*β* (TGF-*β*), vascular endothelial growth factor (VEGF), epidermal growth factor (EGF), insulin-like growth factor-I (IGF-I), fibroblast growth factor (FGF), and hepatocyte growth factor (HGF) (Alsousou et al., 2013). PRP can improve the microenvironment of the degenerative tendon by inducing cell recruitment, proliferation, and differentiation, promoting collagen fiber formation and angiogenesis, and regulating inflammation (Scheme 1). In addition to platelets, PRP contains components such as plasma, leukocytes, and residual erythrocytes, which can synthesize and release some bioactive factors. Neutrophils and monocytes are the most significant leukocytes in PRP. They can release a series of pro-inflammatory mediators, such as peroxidases, matrix metalloproteinases (MMPs), interleukin (IL)-1*β*, IL-6, and tumor necrosis factor-*α* (TNF-*α*), and growth factors, such as platelet-activating factor (PAF), TGF-*β*, VEGF, and EGF (Lyu et al., 2021; Lin et al., 2022). These cytokines can modulate the inflammation of tendinopathy and positively affect tendon healing. PRP can be further divided into leukocyte-poor PRP (LP-PRP) and leukocyte-rich PRP (LR-PRP) according to its composition. LP-PRP has a low platelet content, only 1.5–3 times the baseline level, and contains few erythrocytes and leukocytes (Le et al., 2018). However, LR-PRP usually has high platelet content, 3–8 times the baseline level, and a high leukocyte count (Le et al., 2018).

PRP is derived from autologous whole blood and does not present a risk of immune rejection. A large number of basic experiments have proven that for tendinopathy, PRP has the therapeutic potential to modulate inflammation, stimulate angiogenesis, promote cell proliferation, and increase extracellular matrix synthesis (de Mos et al., 2008; Kajikawa et al., 2008; Zhang and Wang, 2010b; Zhang et al., 2013). However, the clinical efficacy of PRP for tendinopathy is not always satisfactory. Several clinical trials and meta-analyses have shown that PRP therapy does not improve the prognosis of tendinopathy compared with the control groups (Chahal et al., 2012; Saltzman et al., 2016; Filardo et al., 2018). The pathological mechanisms of tendinopathy and the process of tendon healing were reviewed in this article to better understand how PRP affects tendinopathy and explore the reason for the differences in clinical trial outcomes. On this basis, the specific molecular and cellular mechanisms by which PRP manipulates tendon healing were integrated. Finally, we present a critical analysis of the available basic and clinical trial results. We have also pointed out the future direction of basic research and clinical application of PRP in the treatment of tendinopathy, which will play a guiding role in the design of PRP treatment protocols for tendinopathy.

## 2 Physiology and pathology of tendon

### 2.1 Molecular and cellular composition of tendon

Oligovascularity, hypocellularity, and abundant extracellular matrix are unique histological features of the tendon. The tight parallel arrangement of the extracellular matrix within the tendon determines its unique morphology and biomechanical properties. The extracellular matrix mainly includes three types of organic macromolecules: collagen, proteoglycans, and glycoproteins (Citeroni et al., 2020). Collagen is the main substantial component of the tendon extracellular matrix, accounting for 60%–85% of the dry weight of the tendon (Kannus, 2000). Type I collagen is the major component of collagen fibers and accounts for 95% of the total collagen in the tendon (Kannus, 2000). Type I collagen comprises three helical polypeptide chains that initially aggregate to form microfibers. The microfibers further aggregate to form collagen fibers that are considered the basic mechanical transfer unit of the tendon. The collagen fibers eventually aggregate to form a bundle of collagen fibers wrapped by the connective tissue sheaths (i.e., the endotendineum and the epitendineum) (Killian et al., 2012), which form a complete tendon structure. Type III collagen is the second most abundant collagen fibers after type I collagen in the tendon. Type III collagen is small, immature, and unstable; it cannot provide good mechanical support for the tendon (Thorpe and Screen, 2016). Under normal circumstances, type III collagen is mainly found in the endotendineum and epitendineum, but it is present in large amounts in scar tissue formed early in the healing process of the injured tendon (Masuda et al., 2002; Yoshida et al., 2003). The content of type III collagen can be up to 20%–30% in the scar tissue (Masuda et al., 2002; Yoshida et al., 2003). Increased levels of type III collagen relative to type I collagen in the tendon will inhibit collagen fiber growth, causing adhesion and scar tissue formation. This will also reduce the ability of the tendon to transmit stress and increase the risk of tearing. In addition, there are many other types of collagen in the tendon, such as types V, XII, and XIV*.* They play an important role in forming collagen fibers and linking type I collagen to other extracellular matrices (Riley, 2004; Banos et al., 2008). Furthermore, the tendon contains many kinds of proteoglycans adhering closely to collagen. Decorin is a vital component of proteoglycans, accounting for about 80% of the total amount of proteoglycans in the tendon. Decorin helps collagen fibers adapt to the tensile load and plays an important role in tendon development (Thorpe et al., 2013). Other types of proteoglycans, such as biglycan and fibromodulin, exist in small amounts within the tendon. They are combined with type I collagen to form mature collagen fibrils and enhance the mechanical strength of the tendon (Derwin et al., 2001). Proteoglycans carry a large number of negative charges. They can attract and bind to water molecules to form an aqueous gel, affecting the viscoelasticity of the tendon and helping resist compression. Glycoproteins are formed by the glycosylation of proteins, with a similar structure to proteoglycans but with few branching parts. Glycoproteins play a significant role in linking tendon cells to extracellular matrix molecules (O'Brien, 1997). Among glycoproteins, elastin is more common and is usually combined with type I collagen as the main component of elastic fibers. Elastin assists in the storage of elastic potential energy when the tendon is subjected to stress (Kielty et al., 2002). Glycoproteins also include fibronectin, tenascin C, and cartilage oligomeric matrix protein, which interact with collagen or other matrix components to promote tendon repair and improve tendon mechanical stability after injury (Sharma and Maffulli, 2005).

**FIGURE 1:**
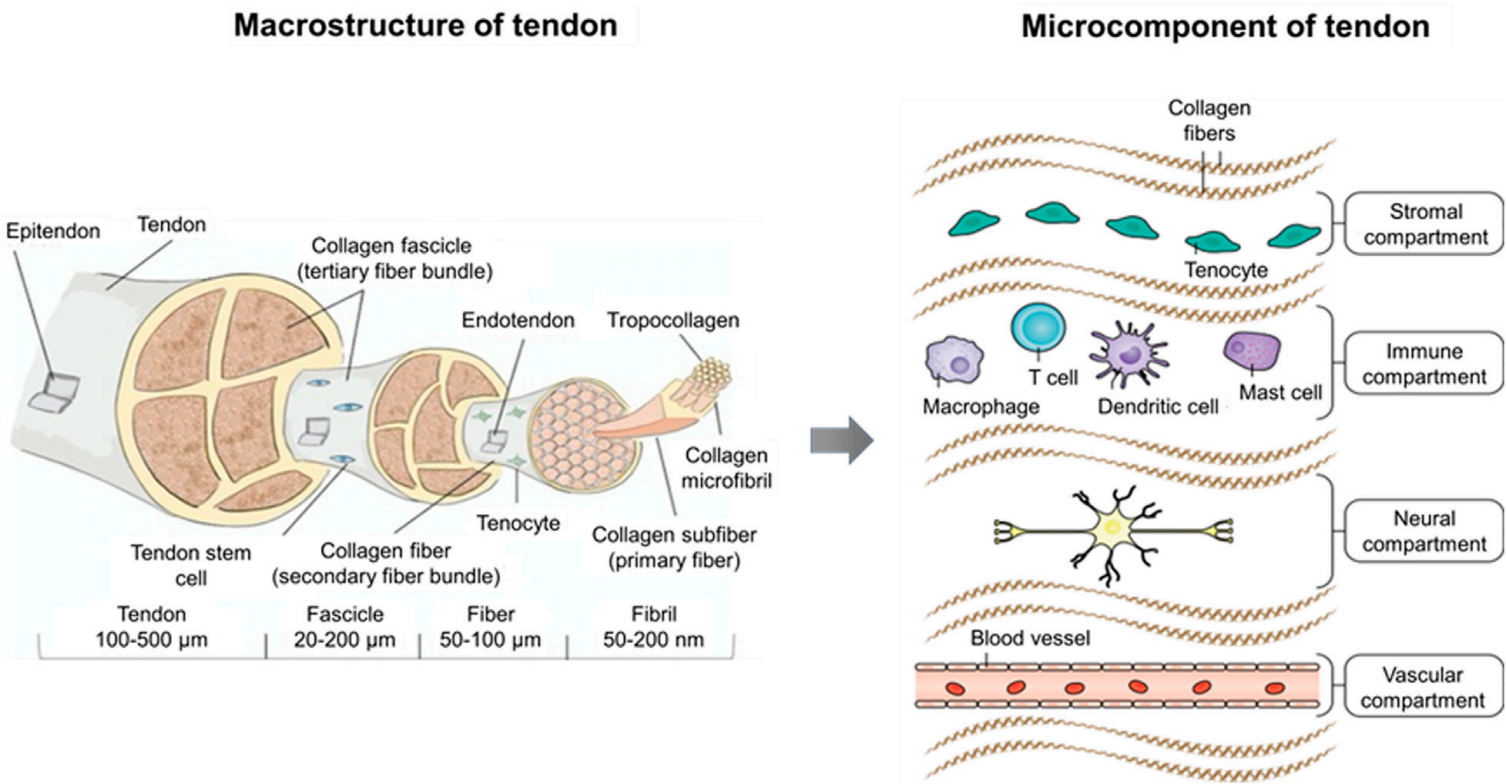
Macrostructure and microcomponent of tendon. The tendon sub-structures (e.g., fascicle, fiber, fibril, and tropocollagen) are illustrated with relative dimensions thereof in a healthy tendon. Microcomponents of the tendon encompass four distinct compartments. The stromal compartment includes tissue-resident tenocytes and matrix components, which are essential for tissue remodeling and repair. The immune compartment involving T cells, dendritic cells, mast cells, and macrophages responds to initial tissue insult through damage-associated molecular patterns (DAMPs) or pathogen-associated molecular patterns (PAMPs). The neural compartment, which in the homeostatic state plays a role in proprioception, interacts with mast cells to modulate adaptive responses in the normal tendon. Although most tendons are poorly vascularized, they respond to hypoxia by secreting angiogenic factors that induce the growth of neovessels, which comprise the vascular compartment (Lipman et al., 2018; Millar et al., 2021).

Tenoblasts and tenocytes are two types of tendon-specific cells, accounting for 90%–95% of all cells in the tendon. They are important for tendon development, extracellular matrix synthesis and turnover, and tendon homeostasis maintenance (Chuen et al., 2004b; Citeroni et al., 2020). Tenoblasts mainly exist in the endotendineum and are in a relatively low differentiation state, with a round appearance and large and oval nucleus. Tenoblasts play a dominant role in tendon development and maturation and can transform into tenocytes (Pennisi, 2002; Chuen et al., 2004a). Tenocytes are derived from the terminal differentiation of tenoblasts and are similar to fibrocytes, with a spindle-shaped appearance, long nucleus, and thin cytoplasm (Russo et al., 2015). Tenocytes are widely distributed within the tendon and colonize among collagen fibers. They precisely regulate the synthesis and breakdown of the extracellular matrix and maintain homeostasis of the tendon. In the case of stress stimulation or tendon injury, tenocytes can mediate tendon repair by increasing the extracellular matrix level through complex signaling pathways (Giordano et al., 2020). In addition, gap junctions between tenocytes enable the exchange of nutrients, signaling molecules, and electrical signals. Thus, tenocytes can coordinate the biological behavior and form a cell communication network within the tendon (McNeilly et al., 1996; Willecke et al., 2002). Bi et al. (2007) discovered the tendon stem cell (TSC), a new type of tendon cell, in the human hamstring tendon. Studies have shown that TSCs have the classic properties of mesenchymal stem cells, such as the presence of specific surface antigens and the ability to self-renew and differentiate into three lineages (i.e., lipogenesis, osteogenesis, and chondrogenesis) (Docheva et al., 2015). Meanwhile, TSCs can express tendon-related genes and form a tendon or tendon-like tissue after *in vivo* implantation (Docheva et al., 2015). The exact role TSC plays in maintaining tendon homeostasis and tendon healing remains uncertain, as well as the exact association among TSCs, tenoblasts, and tenocytes. However, TSCs are still regarded as a potential source of stem cells in tendon healing (Zhang and Wang, 2010a; Zhang et al., 2010; Ruzzini et al., 2014). Additionally, endothelial cells and smooth muscle cells in blood vessels, chondrocytes in insertion sites, and synovial cells in the endotendineum and epitendineum exist within the tendon (Walden et al., 2017) (Figure 1).

### 2.2 Pathogenesis of tendinopathy

**SCHEME 2:**
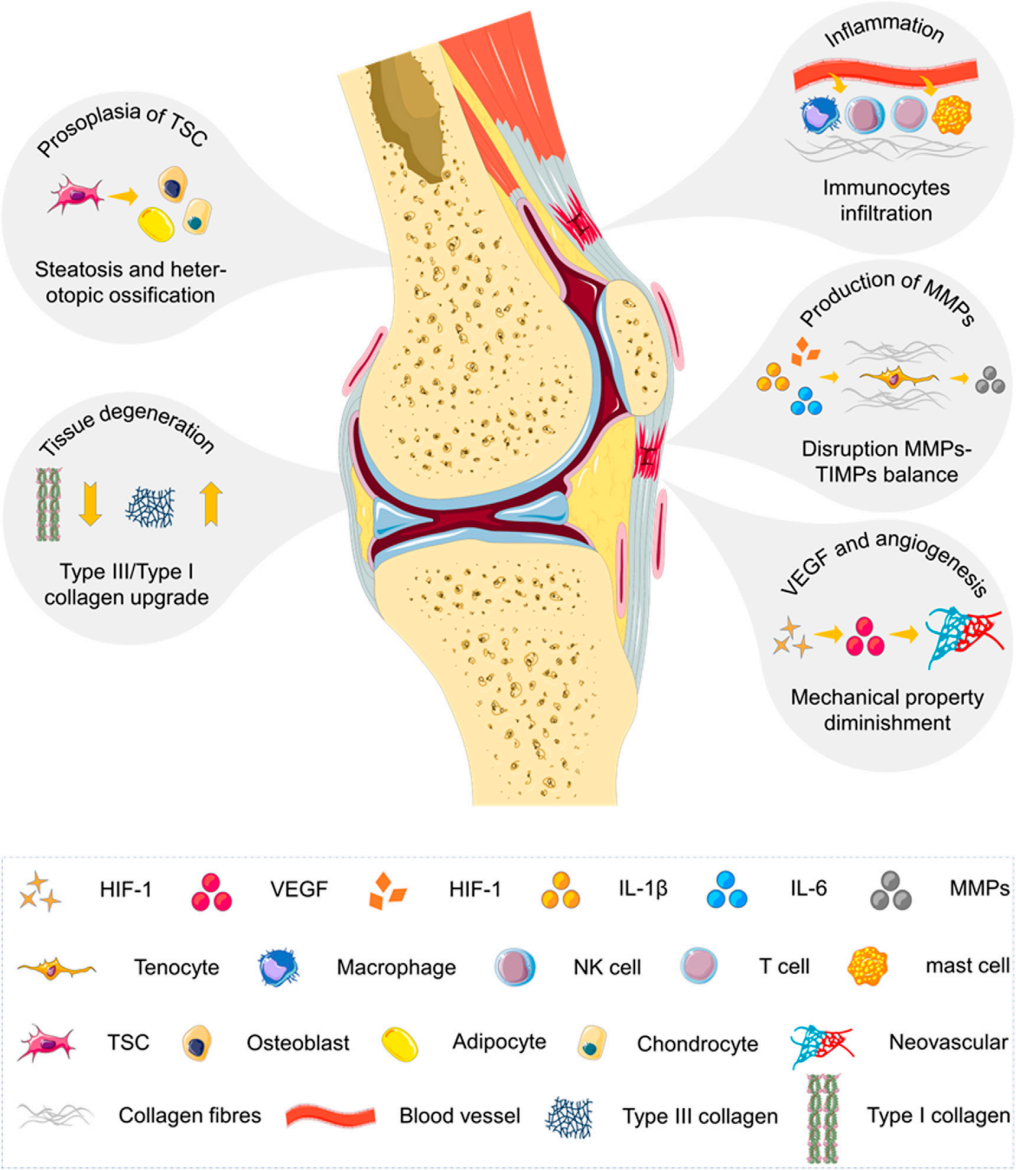
Summarization of underlying biological mechanisms involved in the progression of chronic tendinopathy. Tendinopathy is a medical condition including traumatic or overuse injuries and a spectrum of inflammatory and degenerative tendon changes. The process of angiogenesis and neurogenesis is activated, leading to inflammation and pain. Tendon stem cell receives pathological signals and enters the abnormal differentiation pathway. The balance between matrix metalloproteinases (MMPs) and tissue inhibitors of matrix metalloproteinases (TIMPs) within tendons is also disrupted. The loss of matrix integrity by reduction of total collagen content and augmentation of type III/type I collagen ratio is revealed in pathological tendons. The chronic degenerative tendon has a high level of vascular endothelial growth factor (VEGF) and a large amount of disordered neovascularization attributed to hypoxia and excessive mechanical loading.

Tendinopathy is an injury to a tendon (as from acute trauma or chronic overuse) that is often accompanied by pain, weakness, inflammation, or stiffness (Wu et al., 2017) (Scheme 2). The affected tendons are gray-brown in color, with the original structure destroyed. The matrix components change pathologically. The mechanical properties are weakened. There are clinical features of stiffness, adhesion, functional impairment, local swelling, and pain (Steinmann et al., 2020). Unlike acute tendon injury, which occurs in bursts of intense pain and loss of function, chronic tendinopathy often results in progressive pain and loss of tendon function (Wu et al., 2017). The slow-onset process of tendinopathy may delay the diagnosis of symptoms and ultimately lead to a poor prognosis. With the development of the etiology of tendinopathy, many risk factors associated with tendinopathy have been identified.

The risk factors are generally divided into external, internal, and environmental factors. Different risk factors may be present in different patients, either individually or simultaneously. External factors include tendon overloading, short-term changes in the volume or type of the load, and improper training practices (e.g., the use of inferior equipment or short intervals between repeated loads) (Li and Hua, 2016). Acute loading injury, repeated mechanical injury, and overuse of the tendon caused by external factors are considered the key predisposing factors for tendinopathy (Steinmann et al., 2020). Internal factors related to tendinopathy have obvious individual differences, including age, sex, weight, genetic factors, hormone levels, immune function, and previous medical history (e.g., obesity, hypercholesterolemia, diabetes, hyperlipidemia, and arthritis) (Kjaer et al., 2009; Scott et al., 2015; Leblanc et al., 2017). These internal factors can change the mechanical properties, decrease the load tolerance, and regulate the repair response of the injured tendon. Environmental factors associated with tendinopathy include daily living and working environment, malnutrition, smoking, alcohol abuse, temperature (over low ambient temperature), and pharmacological factors (fluoroquinolones and quinolones, corticosteroid, aromatase inhibitors, and statins) (Scott et al., 2015; Knobloch, 2016).

Tendinopathy-related risk factors will initiate tendinopathy, leading to progressive loss of intrinsic cells and matrix components of the tendon through abnormal cell–cell and cell–matrix communication, eventually resulting in tendon rupture. Angiogenesis and neurogenesis are activated during this process, leading to inflammation and pain (Steinmann et al., 2020). The TSC receives pathological signals and enters the abnormal differentiation pathway, resulting in tendon thickening and calcification (Steinmann et al., 2020). Disruption of the balance between MMPs and the tissue inhibitor of matrix metalloproteinases (TIMPs) plays a key role in the process of tendon degeneration (Jones et al., 2006; Del Buono et al., 2013). The loss of matrix integrity (i.e., reduction in total collagen content and augmentation of type III/type I collagen ratio) is revealed in pathological tendons (Riley et al., 1994a; Riley et al., 1996; Tillander et al., 2002). The chronic degenerative tendon exhibits a high level of VEGF and a large number of disordered neovascular attributed to hypoxia and excessive mechanical loading (Lakemeier et al., 2010; Bosch et al., 2011; Andarawis-Puri et al., 2015).

At present, the main hypothesis of tendinopathy includes degenerative and failed healing pathologies. Degenerative pathology emphasizes apoptosis and matrix degradation of the affected tendon tissue. Failed healing pathology is characterized by an excessive amount of non-collagenous extracellular matrix, collagen fiber disruption, chronic inflammation, and disordered arrangement of neovascularization within the tendon. Cook and Purdam (2009) combined the main ideas of the degenerative pathology hypothesis and the failed healing pathology hypothesis. They proposed that tendinopathy is a continuous process and creatively built a continuum model of tendinopathy (Cook and Purdam, 2009). The continuum model divides the pathological process of tendinopathy from normal tendons to ruptured tendons into three consecutive stages: early reactive tendinopathy, tendon disrepair, and degenerative tendinopathy (Cook and Purdam, 2009). Early reactive tendinopathy, occurring after acute stress or trauma, is characterized by a non-inflammatory proliferative response of cells and matrix. During this period, the tendon attempts to reduce the stress effect per unit area through adaptive and relatively uniform thickening. Tendon disrepair, in which the matrix is severely degraded, is accompanied by a massive increase in the number of cells and the production of new proteins (e.g., proteoglycans and collagen). In the later degenerative tendinopathy, the pathological changes in tendon structure and composition are serious and eventually progress to an irreversible stage. As a result of cell apoptosis, tissue disintegration, severe functional impairment, or even complete failure, the degenerative tendon is prone to rupture. It should be noted that the tendon affected by tendinopathy may simultaneously exhibit different stages of pathological processes. The continuum model lays the foundation for clinicians to assess the pathological progression of tendinopathy and provide targeted therapy for the specific pathological stage. In addition, the model can be continuously evaluated and modified in combination with the researchers’ clinical, histological, and imaging information.

### 2.3 Healing mechanism of injured tendon

The current understanding of the cellular and histologic mechanisms of tendon healing is largely based on animal models with experimentally induced tendon injury (Carpenter and Hankenson, 2004; Warden, 2007). The cellular mechanisms of tendon healing include extrinsic and intrinsic healing mechanisms, which are considered to act synergistically in the process of tissue repair (Kajikawa et al., 2007; James et al., 2008). After a tendon injury, inflammatory cells and fibroblasts from the tendon sheath and blood circulation are attracted to the damaged sites by chemokines, causing cell infiltration and adhesion. Subsequently, the intrinsic cells colonizing the tendon are activated. They migrate to the damaged site, proliferate massively, and express a variety of extracellular matrices. They reconstruct the extracellular matrix and form a vascular network during tendon healing. It is noteworthy that compared with the extrinsic repair mechanism, the intrinsic repair mechanism reduces tissue adhesion and, in a sense, preserves the tendon sliding properties within the sheath (Lipman et al., 2018). This could be attributed to the trait differences in extracellular matrix synthesis and proliferation traits of tenocytes or fibroblasts of different origins (Lipman et al., 2018).

In general, the healing process of the injured tendon can be divided into three main stages in terms of histological characteristics: inflammation, proliferation, and remodeling (Voleti et al., 2012; Andarawis-Puri et al., 2015) (Table 1). These three stages could partially overlap in time and space. The duration of each stage depends on the location and severity of the lesion. Inflammation occurs within 48 h after tendon injury and starts with hematoma formation (Chisari et al., 2021). Activated platelets release clotting factors to initiate the coagulation cascade through degranulation. They also secrete a variety of growth factors and bioactive substances to increase capillary permeability, induce inflammatory cells, and initiate an acute inflammatory response (Kaux and Crielaard, 2013; Marques et al., 2015). Subsequently, the inflammatory cells, including neutrophils, macrophages, and mast cells, migrate to the damaged site, clear the blood clots and necrotic tissue, and further secrete pro-inflammatory cytokines to exacerbate the inflammatory response (Schultz et al., 2003). In addition, platelets and inflammatory cells secrete angiogenic factors that promote angiogenesis and mediate the formation of a disordered neovascular network within the tendon (Tempfer and Traweger, 2015). This is critical for the subsequent healing process and the maintenance of extracellular matrix homeostasis. The damaged tendon enters the proliferation stage after an acute inflammatory response, which is characterized by cellular and extracellular matrix hyperplasia (Nourissat et al., 2013). The proliferation stage lasts for 4–6 weeks. Circulating-derived mesenchymal stem cells, fibroblasts, and TSCs migrate to the affected site and are activated to enter the cell cycle (Chisari et al., 2019). Macrophages convert from type M1 to type M2 and produce large amounts of extracellular matrix together with tenocytes, including collagens and proteoglycans (Li et al., 2021). Much immature and unstable type III collagen is synthesized and forms scar tissue in a disorderly arrangement (Del Buono et al., 2011; Nourissat et al., 2013). Glycosaminoglycan regulates the concentration of water molecules to form local edema within the tendon (Del Buono et al., 2011; Nourissat et al., 2013). At this stage, the tendon exhibits edema, enlargement, a slight increase in mechanical properties, and persistent, intermittent, or activity-related pain (D'Addona et al., 2017). The remodeling stage begins 6–8 weeks after the injury and lasts 1–2 years. This stage is characterized by reduced cell proliferation and extracellular matrix synthesis, replacement of type III collagen with type I collagen, increased cross-linking of collagen fibers and fibrosis, decreased number of capillaries, mature vascular network, and eventual formation of mature tendon tissue (Sharma and Maffulli, 2006). In most patients, especially the middle-aged and elderly, disordered extracellular matrix, impaired collagen fiber integrity, and an increased ratio of type III to type I collagen occur after tendon injury or degeneration. This results in the thickening and hardening of the tendon, with less mechanical strength and mobility than the healthy one.

**TABLE 1:** Tendon repair process in humans.

Phases	Time	Cellular and matrix changes	Cytokines
Inflammatory	48 h	Aggregation of platelets, neutrophils, mononuclear macrophages, erythrocytes, and mesenchymal stem cells, increased pro-inflammatory cytokine production	IL-6, IL-1*β*, TNF-*α*, bFGF, TGF-*β*, PDGF, IGF-I, VEGF
Proliferation (reparative)	6–8 weeks	Increased cellularity and matrix production, activation of local TSCs, promoted type III collagen synthesis	PDGF, TGF-*β*, VEGF, EGF, IGF-I, HGF
Remodeling (consolidation and maturation)	1–2 years	Reduced cellularity and matrix production, reduced type III collagen, increased type I collagen production, maturation of neovascularization	TGF-*β*, IGF-I, GDF-5, GDF-6, and GDF-7

(Schultz et al., 2003; Sharma and Maffulli, 2006; Battery and Maffulli, 2011; Del Buono et al., 2011; Voleti et al., 2012; Kaux and Crielaard, 2013; Nourissat et al., 2013; Docheva et al., 2015; Marques et al., 2015; Chisari et al., 2019; Li et al., 2021).

Tendon healing involves a complex cascade of cytokines. These cytokines originate from platelets, inflammatory cells, endothelium cells, tenocytes, tenoblasts, or TSCs. They are released when stimulated by specific extracellular signals (Citeroni et al., 2020). These cytokines can bind to specific receptors on the target cell membrane and regulate intracellular levels of DNA transcription and translation. Several healing processes, such as cell chemotaxis, proliferation, differentiation, apoptosis, and extracellular matrix turnover, are directly affected (Citeroni et al., 2020). Notably, cytokines play a key role only in the inflammation and proliferation stage. During the inflammation stage, pro-inflammatory cytokines (e.g., IL-6, IL-1*β*, and TNF-*α*) and growth factors (e.g., bFGF, TGF-*β*, PDGF, IGF-I, and VEGF) are mainly released by inflammatory cells and platelets to regulate the early inflammatory response (Battery and Maffulli, 2011; Del Buono et al., 2011). During the reparative stage, growth factors, such as PDGF, TGF-*β*, VEGF, EGF, IGF-I, and HGF, are mainly secreted by the intrinsic cells of the injured tendon to promote proliferation (Voleti et al., 2012). Considering the critical role of cytokines in tendon healing, the administration of exogenous cytokines, such as PRP, has evolved as one of the potential therapies for the treatment of tendon injury.

## 3 PRP-induced regeneration of degenerative tendon

### 3.1 Promotion of tendon cell differentiation and proliferation

After being activated, PRP forms a blood clot that can release a variety of growth factors at high concentrations, including PDGF, TGF-*β*, HGF, FGF, IGF-I, EGF, and high mobility group protein 1 (HMGB1) (Table 2). Therefore, PRP has the function of inducing tendonogenic differentiation of TSCs, promoting the migration and proliferation of TSCs and tenocytes. The binding of PDGF-BB to PDGF receptors can initiate a series of signaling pathways, including Ras-MAPK, PI3K, PLC*γ*, and JAK, impacting chemotaxis, mitosis, and angiogenesis during tendon healing (Evrova and Buschmann, 2017). Specifically, neutrophils and macrophages can be induced to converge on the lesion site and degrade tissue debris by PDGF-BB (Evrova and Buschmann, 2017). Furthermore, PDGF-BB can also stimulate tenocyte migration and proliferation, collagen synthesis, and angiogenesis, thereby improving the mechanical properties of the tendon (Evrova and Buschmann, 2017). The activation of the TGF-*β* signaling pathway can induce the tendonogenic differentiation of TSCs, regulate the metabolism of tenocytes, and stimulate the proliferation of TSCs and tenocytes (Nourissat et al., 2015a). In addition, TGF-*β* is a crucial growth factor that can regulate the production of collagen and other extracellular matrix, which is closely linked to fibrosis and scar formation in tendinopathy (Katzel et al., 2011). In the classical TGF-*β* signaling pathway, TGF-*β* binds to the TGF-*β* type I or type II receptor on the cell surface to form a ligand–receptor complex, activating the downstream Smad protein and regulating gene transcription and translation (Li et al., 2022). Among them, TGF-*β*1 can upregulate the levels of Scleraxis (Scx) and Mohawk (Mkx) genes and induce tendonogenic differentiation of TSC, whereas TGF-*β*2 can stimulate collagen type I alpha 1 (COL1A1) and Scx gene expression (Zhang et al., 2018). Recent studies have shown that TGF-*β* can also transmit signals through various MAPK pathways, collectively known as non-Smad signaling pathways (Zhang, 2017). HGF can promote the proliferation and migration of TSCs by activating HGF/c-Met, MAPK/ERK1/2, and PI3K/AKT signaling pathways (Han et al., 2019). HGF can also hamper the osteogenic differentiation of TSCs by inhibiting the BMP/Smad1/5/8 signaling pathway (Han et al., 2019). It can also improve the biological activity of the tenocyte, regulate the expression of the extracellular matrix, and enhance the biomechanical properties and migration ability of the degenerative tendon (Zhang Z. et al., 2021). Furthermore, bFGF not only induces fibrotic differentiation of circulating mesenchymal stem cells but also stimulates TSC proliferation and accelerates tendon-to-bone healing (Wei et al., 2008; Yuan et al., 2013). *In vitro* studies have shown that IGF-I and EGF can stimulate fibroblast migration and proliferation (Chan et al., 1997; Throm et al., 2010). Zhang J. et al. (2021) showed that in addition to releasing multiple growth factors, PRP can modulate tendinopathy inflammation and induce stem cell migration to injury sites by releasing platelet-derived HMGB1, thereby promoting the healing process. PRP does not provide a single growth factor but rather a proportional combination of multiple growth factors. Therefore, growth factors released by PRP could amplify their therapeutic effects in promoting cell proliferation and differentiation through synergistic actions, but they also could exert adverse effects through antagonism (Docheva et al., 2015). Furthermore, the plasma component of PRP contains fibrinogen and other coagulation factors. The activated PRP can form a temporary fibrin network as a three-dimensional biomaterial scaffold for cell migration, adhesion, and proliferation (Xie et al., 2014).

**TABLE 2:** Growth factors involved in cell recruitment, proliferation, and differentiation.

Growth factors	Proposed function in tendon healing
PDGF-BB	Chemotaxis, mitogenic, and pro-angiogenic induction
TGF-*β*	Promoted collagen synthesis, neovascularization, and fibrosis/excessive scar formation, stimulated TSCs, and tenocyte differentiation and proliferation
HGF	Promoted TSC proliferation, migration, and ECM synthesis, prevented osteogenic differentiation of TSCs
bFGF	Induced fibrogenic differentiation of circulating-derived mesenchymal stem cells and proliferation of TSCs, accelerated tendon-to-bone healing
IGF-I and EGF	Induced migration and proliferation of fibroblasts
Platelet-derived HMGB1	Inflammation regulation and stem cell migration induction

(Chan et al., 1997; Wei et al., 2008; Throm et al., 2010; Katzel et al., 2011; Yuan et al., 2013; Nourissat et al., 2015a; Evrova and Buschmann, 2017; Han et al., 2019; Zhang J. et al., 2021; Zhang Z. et al., 2021).

TSCs play an important role in tendon healing and tendinopathy. Under physiological conditions, TSCs can differentiate into tenocytes and migrate to the injury site to synthesize extracellular matrix. In degenerative tendinopathy, hazardous factors such as excessive mechanical loading, chronic inflammation, and abnormal tissue microenvironment could induce TSCs to differentiate into chondrocytes, osteoblasts, or adipocytes (Zhang X. et al., 2016). Aberrant differentiation of TSCs not only causes tendons to exhibit steatosis or heterotopic ossification, but also highly depletes the tendinous stem cell pool and indirectly reduces the number of tenocytes at the lesion site (Zhang X. et al., 2016). This view is supported by several basic experiments. Compared with TSCs from the healthy tendon, TSCs from the collagenase-induced tendinopathy site exhibit lower expression of tendonogenic markers and higher expression of osteogenic and chondrogenic markers, with lower proliferation and higher senescence rates (Rui et al., 2013). TSCs of biglycan and fibromodulin double-knockout osteoarthritis model mice rather than wild-type mice exhibit decreased expression of tendonogenic markers Scx and type I collagen, increased expression of chondrocyte markers type II collagen and aggrecan, and a significant change in cellular function (Bi et al., 2007). The self-renewal capacity of TSCs from aged/degenerated human Achilles is significantly reduced, but they still retain the potential for multidirectional differentiation (Kohler et al., 2013). The size and adaptive function of the TSC pool from aged/degenerated tendon tissues are remarkably decreased (Kohler et al., 2013). Therefore, adequate and functional TSC is essential for tendon healing, which is one of the key targets of PRP in the treatment of tendinopathy.

**FIGURE 2:**
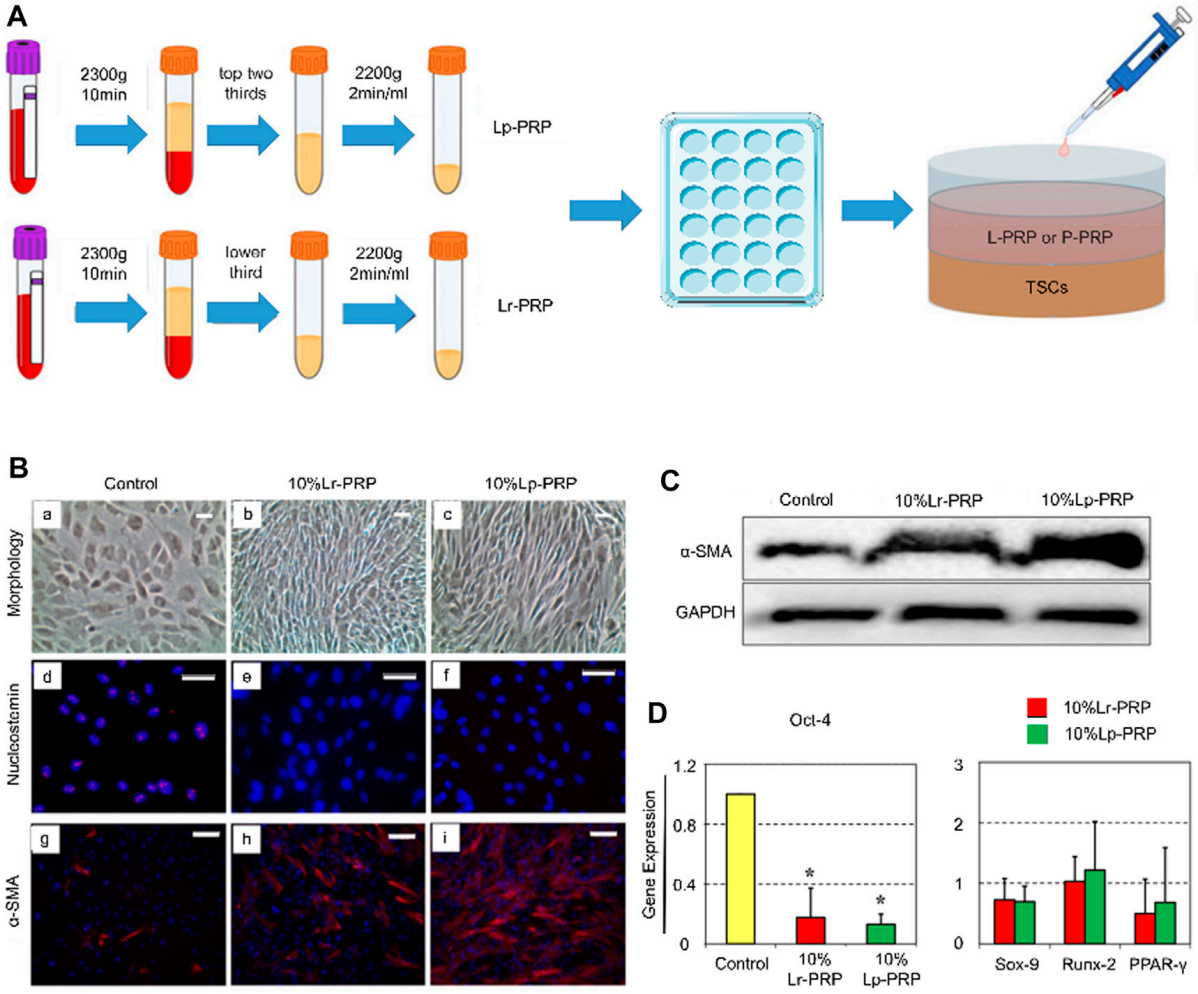
Leukocyte-rich platelet-rich plasma (LR-PRP) and leukocyte-poor platelet-rich plasma (LP-PRP) induce tendon stem cell (TSC) differentiation into tenocytes. **(A)** Illustration of the process of two kinds of PRP and study design. **(B)** Morphology of TSCs in the control, Lp-PRP, and Lr-PRP groups after 14 days in culture **(A–C)**. PRP treatment changed TSC morphology into more elongated tenocyte-like cells and increased the cell number. Immunostaining for nucleostemin and *α*-SMA (d-i). Nucleostemin (a stem cell marker) staining was positive in the control but negative in the PRP-treated cells. PRP treatment increased the expression of *α*-SMA (a marker of active tenocytes) with higher staining. **(C)** Western blot analysis of cultured cells in the control, Lp-PRP, and Lr-PRP groups. An intensely stained *α*-SMA protein band after PRP treatment validated increased the *α*-SMA protein level. **(D)** Expression of the stem cell marker gene Oct-4, Sox-9, Runx-2, and PPAR*γ* of cultured cells in the control, Lp-PRP, and Lr-PRP groups. Oct-4 was reduced in PRP-treated cells. PRP-induced changes in the expression of non-tenocyte genes, Sox-9, Runx-2, and PPAR*γ*, were minimal (Zhou et al., 2015).

Cell culture experiments have shown that PRP-clot releasate (PRCR) could induce the differentiation of TSCs into tenocytes (Zhang and Wang, 2010b). TSCs cultured with PRCR exhibit a fibroblast phenotype, highly expressing tendonogenic genes rather than fat, cartilage, and bone-related genes (Zhang and Wang, 2010b). PRP also substantially increases the number of tenocytes in the culture mediums. These tenocytes highly express *α*-SMA and type I and type III collagen with robust proliferation and collagen-producing potential (Zhang and Wang, 2010b; Zhou et al., 2015) (Figure 2). Flow cytometry analysis has shown that for TSCs cultured in isolation, the administration of PRCR increases the number of type I and III collagen-expressing cells in a dose-dependent manner (Chen et al., 2012). Meanwhile, the number of PPAR*γ*-, SOX-9-, and RUNX2-positive cells decreased (Chen et al., 2012). This suggests that PRCR accelerates tendon healing by stimulating the tendonogenic differentiation of TSCs and promoting extracellular matrix synthesis. At the same time, PRCR has the potential to inhibit the differentiation of TSCs into adipocytes, chondrocytes, and osteocytes. Based on this, Zhang and Wang (2014) further explored the condition and mechanism of PRCR in inhibiting the abnormal differentiation of TSCs. They found that the aberrant differentiation of TSCs was significantly inhibited when cultured in 10% (volume/volume) PRCR without any pretreatment. However, for TSCs pretreatment with non-tendonogenic media for 2 days, PRCR could only significantly inhibit the chondrogenic differentiation and slightly inhibit adipogenic and osteogenic differentiation. Finally, PRCR failed to reverse the abnormal differentiation of TSCs pretreated with non-tendonogenic media for 1 week. These results demonstrate that PRP cannot prevent the development of tendinopathy in the later stage, although PRP does not worsen the degenerative process. This might partially explain why PRP is not clinically effective in some patients with refractory tendinopathy. In this case, thorough tissue debridement is necessary to improve the lesion site microenvironment and induce TSCs to re-enter the tendonogenic differentiation pathway (Witt and Hyer, 2012; Gill et al., 2013). Xu et al. (2017) compared PRP–collagen–TSC constructs (PCTCs) and collagen–TSC constructs (CTCs). They found that the microstructure of PCTCs displayed more obvious microvascular and fibrous tissue characteristics than CTCs after 3 weeks in culture. After *in vivo* transplantation, PCTCs improved the macroscopic structure, histological characteristics, and biomechanical strength of ruptured Achilles in comparison with CTCs, with a better effect on tendon healing. Likewise, Imai et al. (2019) investigated the effects of PRP on peritendinous cells and reported that PRP could promote the migration and proliferation of peritendinous cells and induce the expression of the tendonogenic markers Scx and COL1A1.

Recently, researchers have proposed the combination of PRP and TSCs for the treatment of tendinopathy (Wang and Nirmala, 2016). PRP can provide abundant growth factors and a collagen scaffold, which can be a good biomaterial for TSC delivery. Exogenous TSCs can replenish the TSC pool and accelerate the healing process. For example, Chen et al. (2014) demonstrated that the combination therapy of PRP and TSCs promoted healing quality and improved histological and biomechanical scores in collagenase-induced Achilles tendinopathy. Meanwhile, the mRNA and protein expression levels of tendonogenic genes (type I collagen, Scx, and Tenascin C) increased (Chen et al., 2014). The FAK and ERK1/2 signaling pathway plays a key role (Chen et al., 2014).

Tenocytes are a major cellular component of the tendon and play an important role in maintaining its homeostasis. Meanwhile, they can proliferate and synthesize extracellular matrix to initiate the tendon repair process (Zhou and Wang, 2016). Anitua et al. (2005) reported that 20% PRP (volume/volume) could induce the synthesis of VEGF and HGF in tenocytes and promote tenocyte proliferation. de Mos et al. (2008) found that PRCR, compared with PPCR, further promoted tenocyte proliferation and collagen synthesis and increased the concentration of PDGF-BB, VEGF, and TGF-*β*1 in the medium. *In vitro* experiments designed by Wang et al. (2012) showed similar results. They reported that tenocytes were activated, proliferated, and collagen synthesis was stimulated by 10% (volume/volume) PRCR. Furthermore, the mRNA expression levels of Scx, type I collagen, type III collagen, and decorin were decreased, whereas the differentiation potential of tenocytes was preserved by PRCR (Wang et al., 2012). However, the tenocyte samples used in these studies were all from healthy tendons and might not fully reflect the effect of PRP on the tenocytes harvested in tendinopathy tendons. On this basis, Jo et al. (2012) investigated the effect of PRP on tenocytes from the diseased rotator cuff. They found that PRP could stimulate the proliferation of tenocytes in a dose-dependent manner; induce the expression of Scx, decorin, and tenascin C; promote the synthesis of type I and type III collagen; and increase the total amount of collagen (Jo et al., 2012). Pauly et al. (2018) also isolated tenocytes from patients with rotator cuff tendinopathy and cultured them in a medium containing autologous PRP. Their results showed that PRP increased the concentration of growth factors, such as IGF-I, TGF-*β*1, and PDGF-AB, in the medium compared with the control group and promoted cell proliferation and the absolute synthesis of type I collagen. Furthermore, Yoon et al. (2018) evaluated the effect of 10% (volume/volume) PRP on tenocytes from the healthy rotator cuff and the degenerative torn rotator cuff. They reported that PRP could promote the proliferation and extracellular matrix synthesis of tenocytes from both sources. However, tenocytes from the diseased rotator cuff had higher rates of cell proliferation and glycosaminoglycan synthesis, as well as a higher ratio of type I/type III collagen (Yoon et al., 2018). There are few studies on the exact mechanism of PRP in promoting tenocyte proliferation. Yu et al. (2015) reported that PRP, which contains high concentrations of TGF-*β*1 and PDGF, could modulate the Stat3/p27(Kip1) activity, promote the expression of cyclin–Cdk complexes, and, in turn, stimulate tenocyte proliferation.

In addition to directly promoting cell differentiation and proliferation, PRP can indirectly increase the number of cells in the lesion site by inhibiting the apoptosis of tenocytes. Platelet microparticles are tissue fragments of 0.1–1 μm in diameter, secreted by activated, stressed, or apoptotic platelets. They have been shown to enhance cell viability by activating protein kinase B through phosphorylation to inactivate B-cell lymphoma-2 (Bcl-2) associated death promoter, a member of the Bcl-2 family with pro-apoptotic effects (Burnouf et al., 2014). PRP-releasing substances, including HGF, stromal cell-derived factor-1 alpha (SDF-1α), 5-hydroxytryptamine, ADP, sphingosine-1-phosphate (S1P), IGF-I, brain-derived neurotrophic factor (BDNF), and TIMP-1, can also resist apoptosis (Gawaz and Vogel, 2013). Recently, Yu et al. (2021) evaluated the therapeutic effects of PRP-releasing substance on an injured Achilles model in rats. The results showed that the PRP-releasing substance could reduce the number of apoptotic cells at the edge of injury, promote the synthesis of collagen, and reduce the infiltration of macrophages. Compared with the control group, the rat Achilles treated with PRP could bear further mechanical loading.

The anti-aging gene Sirtuin 1 (Sirt1) is a nicotinamide adenine dinucleotide-dependent class III histone deacetylase that targets transcription factors to adapt gene expression to metabolic activity. Sirt1 is involved in telomerase reverse transcriptase and genomic DNA repair, with its participation in telomere maintenance for chromosome stability and cell proliferation. Sirt1 is also involved in cell cycle regulation, cell differentiation, cell survival, and apoptosis, with effects on nonalcoholic fatty liver disease, inflammation, energy metabolism, cognition, glucose/cholesterol metabolism, and amyloidosis. Metabolic diseases profoundly interfere with tendon health, including type 2 diabetes mellitus, dyslipidemia, chronic kidney disease, and nonalcoholic fatty liver disease (Afifi et al., 2019; Turk et al., 2020; Lin et al., 2023). For instance, chronic liver disease raises the risk of tendon disorder around 1.33-fold higher than usual (Lin et al., 2023). Tendon disorder occurs on average 3 years after being diagnosed with liver disease (Lin et al., 2023). The risk of tendon disorder increases further in this population with concurrent use of certain tendon–toxic medications, such as systemic glucocorticoids and statins (Lin et al., 2023). Therefore, Sirt1 activation is important for reversing metabolic diseases, such as nonalcoholic fatty liver disease, with relevance to tendinopathy. In addition, Simic et al. (2013) showed that Sirt1 deacetylated Runx2 and *β*-catenin to regulate the differentiation of mesenchymal stem cells. Sirt1 activated by resveratrol was reported to promote the osteogenic differentiation of mesenchymal stem cells by increasing Runx2 expression, facilitating its combination with PPARγ, and inhibiting the activity of PPARγ through its cofactor nuclear receptor, co-repressor 1 (Hata et al., 2003). Overexpressed Sirt1 can be anchored to NCoR1 and NCoR2, the inhibitory co-factors of PPAR*γ*, which in turn suppress the expression of PPAR*γ* and C/EBP*α* and further reduce the transformation of pre-adipocytes into adipocytes (Hata et al., 2003). In TSCs, Sirt1 is demonstrated to time-dependently promote the osteogenic differentiation by upregulating *β*-catenin and Runx2 and to inhibit the adipogenic differentiation by inhibiting the PI3K/AKT pathway with the downregulation of CEBP*α* and PPAR*γ* (Liu et al., 2016). During the repair process of tendon injury, TSCs favor differentiation into cartilage and bone, whereas the accumulation of adipose tissue is not conducive to recovery from tendinopathy (Liu et al., 2016). Therefore, by targeting Sirt1, it may be possible to regulate the osteogenic differentiation of TSCs and adipose accumulation in injured tendons. Furthermore, Sirt1 downregulation leads to similar effects caused by stimulation with IL-1*β*, such as enhanced inflammatory signaling, reduced cell survival, and activated NF-*κ*B in human tenocytes (Busch et al., 2012). In contrast, Sirt1 activation by resveratrol suppresses IL-1*β*-induced inflammatory signaling and apoptosis through deacetylation of NF-*κ*B subunit p65 and tumor suppressor p53, thereby inhibiting the activation pathway of NF-*κ*B- and p53-mediated apoptosis of tenocytes (Busch et al., 2012). Prevention of adhesion is associated with Sirt1 signaling. An *in vitro* experiment conducted by Chen et al. (2015) demonstrated a regulatory role of Sirt1 signaling (via NF-*κ*B, a subunit of p53, and p53) in the prevention of adhesion using chitosan. These results suggest that the upregulation of Sirt1 appears to be useful for the treatment of tendinopathy.

PRP has the potential to activate the Sirt1 signaling pathway, promote tenocyte proliferation, regulate TSC differentiation and inflammation, and prevent tendon adhesion (Li et al., 2021). Weng et al. (2021) comprehensively investigated PRP effects on the metabolic reprogramming of fibroblasts and demonstrated that PRP halts the senescence progression of fibroblasts by activating Sirt1 expression. In addition, thrombin-activated PRP can increase the expression of Sirt1 in periodontal ligament stem cells and significantly enhance cell viability, ALP activity, osteogenic-related mRNA levels, and alizarin red-mineralization activity in a dose-dependent manner (Xu et al., 2021). Further studies are needed to confirm the exact role and specific mechanism of PRP in activating the Sirt1 signaling pathway in TSCs and tenocytes of chronic tendinopathy.

### 3.2 Promotion of tendon matrix synthesis and remodeling

In recent decades, biochemical and molecular biological studies of tendinopathy have deepened our understanding of the process of tendon degeneration. Tendinopathy can be considered an abnormal matrix remodeling process of the tendon. Excessive mechanical loading disrupts the balance between matrix synthesis and catabolism (Steinmann et al., 2020). Several studies have examined the changes in matrix molecules in the tendons affected by chronic tendinopathy (Riley et al., 1994a; b; Riley et al., 1996; Riley, 2005; Yamada et al., 2007). The total amount of collagen in pathological tendon tissue significantly decreases. The levels of type I and type II collagen and the ratio of type III collagen to type I collagen increase (Riley et al., 1994a; Riley et al., 1996; Tillander et al., 2002). For proteoglycans, some studies suggested that the contents of hyaluronic acid, glycosaminoglycan, and aggrecan increased remarkedly, whereas the content of decorin decreased significantly (Riley et al., 1994a; Lo et al., 2005). In addition, glycoproteins, such as tenascin C and fibronectin, generally increase in the degenerative tendon (Riley et al., 1996; Tillander et al., 2002). The activity of various MMPs also changes. There is an increase in the levels of MMP-1, MMP-2, MMP-23, disintegrin metalloproteinase 12 (ADAM-12), and platelet-reactive protein disintegrin metallopeptidase 2 (ADAM-TS2) and -TS3, but a decrease in MMP-3, MMP-10, MMP-12, and MMP-27 levels (Riley, 2008). Disruption of the balance between MMPs and TIMPs plays a key role in the process of tendon degeneration (Jones et al., 2006; Del Buono et al., 2013). Tendons can adapt to mechanical loading by increasing collagen synthesis and the activity of multiple MMPs. This adaptation enhances the mechanical strength and the viscoelastic properties of the tendon and reduces the stress sensitivity of the tendon, generally enhancing its load resistance. However, long-term repeated excessive mechanical loading leads to micro-injury accumulation in the tendon, which promotes the progression of tendinopathy and eventually leads to rupture (Diniz-Fernandes et al., 2018).

PRP contains many growth factors, such as IGF-I, FGF, TGF-*β*, EGF, HGF, and PDGF, which can prevent the change in the matrix molecule caused by tendinopathy to some extent. IGF-I plays an important regulatory role in collagen synthesis. IGF-I upregulation can stimulate collagen synthesis by activating downstream signal transduction pathways, such as ERK and Akt/mTOR (Hansen et al., 2013; Ren and Anversa, 2015). Local injection of IGF-I can promote collagen synthesis and increase the diameter of collagen fibers in healthy tendon tissues. This can also reduce swelling, promote tenocyte proliferation, and increase the total content of collagen in the degenerative tendon (Olesen et al., 2006; Hansen et al., 2013; Nielsen et al., 2014). bFGF can stimulate the proliferation and migration of tenocytes, upregulate the levels of type I and III collagen, and coordinate the orientation of collagen fibers, thereby improving the mechanical properties of the tendons (Goncalves et al., 2013; Chen et al., 2021). TGF-*β* is a crucial growth factor that regulates the production of the extracellular matrix. TGF-*β*2 can stimulate the expression of Col1A1 and Scx genes. A moderate amount of TGF-*β*2 can promote tendon healing, but excessive TGF-*β*2 is closely related to fibrosis and scar formation of the injured tendon (Katzel et al., 2011; Zhang et al., 2018). EGF has been shown to accelerate wound healing by promoting the formation of extracellular matrix and granulation tissue and stimulating fibroblast proliferation and tissue regrowth (Throm et al., 2010).

Numerous *in vitro* experiments have proven that PRP can stimulate tenocytes derived from normal or diseased tendons to synthesize non-collagen extracellular matrices and increase the total amount of collagen and the expression of type I and III collagen (Jo et al., 2012; de Vos, 2016; Kelly et al., 2016; Pauly et al., 2018; Yoon et al., 2018). In addition, PRP did not appear to affect the level of type I/III collagen expression in tenocytes (de Mos et al., 2008; Jo et al., 2012). Notably, de Mos et al. (2008) reported that PRP reduced the relative cellular expression of type I and III collagen (i.e., the ability of an individual tenocyte to synthesize type I and III collagen). However, owing to the increase in the number of tenocytes, the total amount of collagen remained higher than that of the control group (de Mos et al., 2008). This conclusion was confirmed by previous studies (Wang et al., 2012; Pauly et al., 2018). In contrast, some studies did not support this view (Jo et al., 2012; Kelly et al., 2016; Yoon et al., 2018). A variety of factors can cause differences in the collagen expression capacity of tenocyte samples in different experiments, including the age of donors, the origin of tenocytes, the pathological changes in the tendon tissue, and the components of PRP.

**FIGURE 3:**
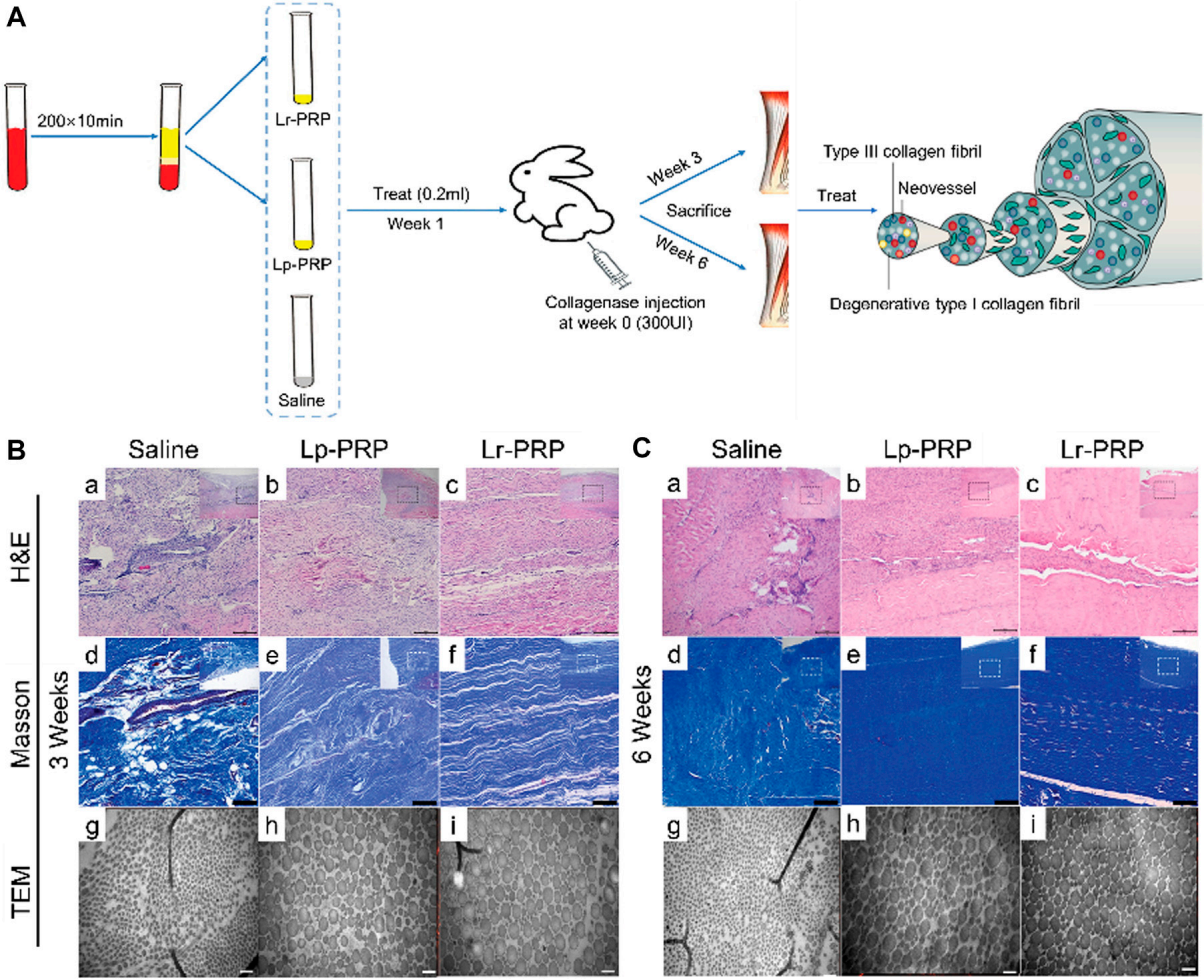
Effects of leukocyte-rich platelet-rich plasma (LR-PRP) and leukocyte-poor platelet-rich plasma (LP-PRP) on Achilles tendinopathy when applied at an early stage. **(A)** Illustration of the process of two kinds of PRP and study design. **(B)** Hematoxylin and eosin (H&E) staining, Masson staining, and transmission electron microscopy (TEM) of the saline, Lp-PRP, and Lr-PRP groups at 3 weeks. A better fiber structure and less angiogenesis were observed in the Lr-PRP and Lp-PRP groups. **(C)** H&E staining, Masson staining, and TEM of the saline, Lp-PRP, and Lr-PRP groups at 6 weeks. The tendons in both treated groups had better recovery than the saline group, but they were still worse compared with the healthy tendons (Jiang et al., 2020).

The therapeutic effect of PRP in promoting matrix synthesis was also validated *in vivo* and manifested macroscopically as an improvement in the biomechanical properties of the tendon and a shorter recovery time. Yu et al. (2021) investigated the therapeutic effect of PRP on rat models of acute Achilles injury. They found that PRP could increase the number of tenocytes in the vicinity of the wound and stimulate collagen synthesis in a short period, thus increasing the maximum load the tendon can bear and shortening the recovery time. Some studies evaluated the long-term therapeutic effect of PRF or PRP on a rabbit model with Achilles injury using imaging and histological analysis (Fukawa et al., 2015; Wong et al., 2020). Meanwhile, the release kinetics of growth factors in PRF was tested *in vitro* (Fukawa et al., 2015; Wong et al., 2020). The results show that PRF has good properties of growth factor delivery, which could store and stably release PDGF, bFGF, TGF-*β*1, and IGF-I for a long time. Ultrasound images suggest that the area of the hyperechoic zone (consisting of scar tissue formed by a disordered arrangement of cells and matrix) in the rabbit Achilles with PRF implantation is smaller than that in the control group. MRI suggests that the T2 signal of the rabbit Achilles in the PRP treatment group decreases gradually over time, reflecting the normalization of collagen fiber arrangement and the depletion of water concentration. Histological analysis shows that tendons in the PRF group have thicker, denser, and more continuous collagen fibers arranged in the direction of stress and have a smaller degenerative area with bone and cartilage. A basic experiment using histological analysis to assess the effect of PRP on Achilles injuries in rabbits came to a similar conclusion (Takamura et al., 2017). In the first 2 weeks after surgery, PRP could promote the migration and proliferation of tenocytes and stimulate angiogenesis. In the next 4 weeks, PRP can promote collagen synthesis and tissue maturation, thereby accelerating the healing process of the tendon. In conclusion, PRP can improve the mechanical property and load-bearing capacity of injured Achilles, shorten the healing time, and promote tendon maturity. This is clinically beneficial because PRP not only reduces the risk of secondary rupture of the Achilles, but also promotes the early recovery of motor function. Early moderate weight-bearing and exercise can prevent adhesion, extend the motion range of the affected limb, and accelerate recovery, achieving higher patient satisfaction (Maffulli et al., 2003). On this basis, Alsousou et al. (2015) treated 20 patients with Achilles rupture by PRP and performed histological and immunochemical biopsies 6 weeks after surgery. The results show that local application of PRP can promote the deposition of type I collagen, increase the ratio of type I collagen to type III collagen, and upregulate the content of glycosaminoglycan. It can also promote the recovery and maturity of the injured tendon, reflected in fewer blood vessels, a smaller proportion of cells, and a more consistent and regular arrangement of collagen fibers. For rotator cuff tendinopathy model animals, PRP also has a good therapeutic effect. Some studies have shown that the administration of PRP can increase collagen content, promote tissue maturation, and improve the biomechanical properties of the tendon, including stiffness, tenacity, and mechanical load capacity (Hapa et al., 2012; Dolkart et al., 2014; Ersen et al., 2014). Kobayashi et al. (2020) reported the therapeutic effect of PRP on the patellar tendon injury model in mice. According to the Bonar Score, injured patellar tendons in the PRP group form more neovascularization in the early stage of the healing process and have more aligned collagen fibers in the late stage of the healing process, which indicates that PRP can accelerate the healing of injured patellar tendons (Kobayashi et al., 2020). In addition to treating the animal model with a tendon injury, PRP can also improve the healing of degenerative tendons induced by collagenase, increase the expression of type I collagen protein, inhibit the process of degeneration in a certain sense, and promote tissue remodeling and maturity (Moshiri et al., 2014; Yan et al., 2017; Jiang et al., 2020) (Figure 3).

Recently, several basic studies have reported that PRP might play a role in promoting MMP secretion by tenocytes or TSCs. For example, PRP or PRCR could significantly increase the mRNA and protein levels of type I collagen and MMP-1 in human fibroblasts (Kim et al., 2011; Shin et al., 2014). Pifer et al. (2014) showed that certain concentrations of MMP-2, MMP-3, and MMP-9 could be detected both in low platelet concentration LP-PRP (prepared by ACP) and in high platelet concentration LR-PRP (prepared by GPS). Most of these MMPs are in an active state and can maintain their plasma levels for at least 6 days. Human ligament fibroblasts also release a variety of MMPs after exposure to ACP or GPS-prepared PRP (Pifer et al., 2014). In addition, Zhou et al. (2015) found that tenocytes treated with LR-PRP could express more catabolic genes, including MMP-1, MMP-13, IL-1*β*, IL-6, and TNF-*α*. The research implicated that PRP might regulate the tissue remodeling process by controlling the level of MMPs within the tendon. The balance between MMPs and TIMPs plays a key role in tendon remodeling (Jones et al., 2006; Del Buono et al., 2013). A moderate amount of MMPs can reduce hypertrophy and scar tissue formation of the affected tendon and promote matrix remodeling (Fleming et al., 2009; Scollon-Grieve and Malanga, 2011; Thanasas et al., 2011). Excessive MMPs promote catabolism and aggravate the degenerative process in tendinopathy (Marqueti et al., 2006; Arnoczky et al., 2007; Bedi et al., 2010). Currently, only a few studies have explored the efficacy of PRP in combination with MMP inhibitors in the treatment of tendinopathy. For example, Jafari et al. (2019) compared the efficacy of single PRP *versus* PRP plus broad-spectrum or narrow-spectrum MMP inhibitors for tendinopathy. They found that tendons treated with MMP narrow-spectrum inhibitor (specific inhibition of MMP-13) and PRP had the best histological and biomechanical properties.

### 3.3 Regulation of tendon angiogenesis

The role of VEGF and angiogenesis in tendinopathy or tendon healing has not been fully elucidated. The present studies suggest that VEGF and neovascularization may play different roles in different stages of tendinopathy. After an acute tendon injury, hypoxia, pro-inflammatory cytokines, nerve signals, and mechanical loading can upregulate the VEGF level. Increased metabolism attributed to tendon injury indirectly leads to hypoxia and upregulation of the hypoxia-inducible factor-1 (HIF-1) level (Pufe et al., 2005; Li et al., 2013; Halper, 2014; Rahim et al., 2016). HIF-1 can further induce VEGF gene transcription. The release of pro-inflammatory cytokines, such as IL-1*β*, IL-6, and IL-8, and the infiltration of inflammatory cells can also promote VEGF synthesis. Upregulated nerve growth factor promotes neuronal growth, leading to an increased level of neurogenic VEGF (Pufe et al., 2005; Li et al., 2013; Halper, 2014; Rahim et al., 2016). Excessive mechanical loading can also promote the release of VEGF from the tendon tissue. The trend of VEGF concentration is similar in various animal models with tendon injury. This increases gradually in the early stage of the proliferative phase and eventually decreases with the relief of symptoms (Liu et al., 2021b). VEGF is a potent angiogenic stimulant. It can promote angiogenesis and increase vascular permeability, mediating the delivery of circulating cells, growth factors, oxygen, and nutrients to the tendon lesions (Molloy et al., 2003). In addition, neurogenic VEGF-mediated neovascularization is surrounded by perivascular cells. This cell type has mesenchymal stem cell properties and is considered a source of TSCs, which can mediate the healing process of the injured tendon (Wu et al., 2017; Li et al., 2019; Lee et al., 2021). Therefore, the changes in VEGF and angiogenesis reflect the early healing process of acute tendon injury in a sense.

Some researchers believe that PRP promotes vascular endothelial cell proliferation and augments vasopermeability in the early stage of tendinopathy, providing a suitable microenvironment for tendon healing. Activated platelets release a range of pro-angiogenic factors, such as VEGF, HGF, TGF-β, PDGF-BB, IL-8, angiopoietin, and chemokine (C-X-C motif) ligand 12 (CXCL12), as well as MMP-1, MMP-2, and MMP-9 (Nurden et al., 2008). Leukocytes further replenish the pro-angiogenic protein pool of PRP by releasing VEGF, angiopoietin, FGF, HGF, PDGF, and MMPs (Kobayashi et al., 2016; Andia and Abate, 2018). As one of the most important angiogenic factors, VEGF can regulate angiogenesis in both healthy and degenerative tendons (Molloy et al., 2003; Pufe et al., 2005). VEGF is a specific mitogen of endothelial cells that promotes the growth and proliferation of endothelial cells and perivascular cells, increases the permeability of capillaries, and promotes angiogenesis within the tendon (Wu et al., 2017; Peach et al., 2018). Meanwhile, VEGF has the function of promoting the proliferation of fibroblasts, stimulating the chemotaxis of macrophages and granulocytes, and initiating the production of other growth factors (Liu et al., 2021a). bFGF has a strong angiogenic effect in synergy with VEGF, which can regulate intratendon inflammation, cell proliferation, angiogenesis, and collagen synthesis (Thomopoulos et al., 2010; Tang et al., 2016). PDGF-BB can indirectly regulate the migration of endothelial cells and vascular smooth muscle cells and participate in the maturation and stabilization of neovascularization by upregulating the levels of VEGF and integrin (Evrova and Buschmann, 2017). Furthermore, MMPs are directly involved in the process of angiogenesis. MMPs can regulate the activity of chemokines, such as IL-8 and CXCL12, as well as growth factors, such as TGF-*β* and HGF, released by PRP (Moser et al., 2004; Kawase et al., 2015). MMPs can also stimulate the migration of endothelial cells by degrading the extracellular matrix (Andia and Abate, 2018). Moreover, MMP-2 and MMP-9 can activate the wnt/*β*-catenin signaling pathway, which is beneficial to the survival and proliferation of endothelial cells (Kawase et al., 2015).

Basic research further confirms that PRP has the therapeutic effect of promoting angiogenesis in the initial period of tendon healing. Bosch et al. (2011) used color Doppler ultrasound and Factor VIII immunohistochemical staining to study the effect of PRP on the angiogenesis of acutely injured tendons. They found that PRP significantly increased angiogenesis for at least 23 weeks compared with the placebo (Bosch et al., 2011). The author speculated that a a large number of neovascular vessels within the tendon might be one of the main factors that a single PRP injection can promote the long-term repair of the injured tendon (Bosch et al., 2011). Kobayashi et al. (2020) explored the specific mechanisms by which PRP affects tendon healing using a patellar tendon injury mouse model. As a result, the Bonar vascular score of the PRP group was significantly higher than that of the control group 2 and 4 weeks after the operation. Kobayashi et al. also reported that PRP not only had a direct effect of stimulating tenocytes to produce an extracellular matrix, but also indirectly facilitated tendon healing by promoting the angiogenesis and the recruitment of circulating repair cells (Kobayashi et al., 2020). These results suggest that the pro-angiogenic proteins of PRP can effectively mediate angiogenesis in the early stage of tendon healing and provide adequate blood supply and a suitable microenvironment for tendon repair. PRP also has a good therapeutic effect on injured Achilles of New Zealand white rabbits (Lyras et al., 2009). The injured Achilles in the PRP group had more neovascularization than the control group in the first 2 weeks (Lyras et al., 2009). Although at the 4th week, in the PRP group, the number of neovascular vessels and the rate of tenocyte metabolism significantly decreased, the consistency of collagen fiber arrangement and the histological characteristics of tendons were improved (Lyras et al., 2009). Additionally, *in vitro* experiments have reported that PRP can temporarily improve cell migration by activating VEGF receptor 2 born on the endothelial cell membrane directly (Kawase et al., 2015). However, this therapeutic effect is dose- and time-dependent and cannot be sustained over the long term, which is attributed to the reduced sensitivity of endothelium to PRP over a long period (Kawase et al., 2015). This is also an important factor to be considered when designing similar experiments.

Under normal circumstances, neovascularization in the injured tendon will gradually subside as the healing process progresses, eventually forming a mature vascular network. However, chronic tendinopathy can lead to the long-term presence of intratendon capillaries and worsen the microenvironment of tendon healing (Liu et al., 2021b). The VEGF-mediated angiogenesis cascade appears to be highly active in the chronic degenerative tendon attributed to prolonged hypoxia and excessive mechanical loading (Lakemeier et al., 2010; Bosch et al., 2011; Andarawis-Puri et al., 2015). However, Jarvinen (2020) reported in a recent study that neovascularization within the chronic degenerative tendon is hyperpermeable, unable to provide oxygen and nutrients to the tissue and transport metabolic waste. This means that neovascularization induced by tendinopathy is weak or even non-functional, without normal blood flow perfusion, and unable to change the hypoxic state of the affected areas. Fibrin-rich exudates constantly leak from the neovascular, leading to fibrin degeneration of the tendon (Jarvinen, 2020). This is a typical histological feature of tendinopathy. The persistent vascularization in tendinopathy might be attributed to low-grade inflammation triggered by chronic irritants and might be considered a symbol of incomplete repair (Bosch et al., 2011). High levels of VEGF in the affected tendon can exacerbate the side effects of vascularization, resulting in constant pain and decreased biomechanical function of the tendon (Sahin et al., 2012; Korntner et al., 2019). Neovascularization and nerve fibers usually form together. The latter can secrete a series of neurotransmitters, such as calcitonin gene-related peptide and substance P, eventually causing long-term pain in the affected tendon (Ackermann et al., 2003). Meanwhile, a high level of VEGF can stimulate the expression of MMPs and inhibit the expression of TIMPs in endothelial cells and tenocytes, disrupting the balance of extracellular matrix remodeling and impairing the mechanical properties of the tendon (Sahin et al., 2012; Halper, 2014). In addition, long-term hypoxia and overexpression of VEGF are considered the key inducers of neovascularization, which is hyperpermeable, immature, and non-perfusionable (Jarvinen, 2020). These anomalous vascular networks cannot alter hypoxia and nutrient deficiencies of the affected tendon (Jarvinen, 2020). High levels of VEGF have also been shown to maintain inflammation in tendinopathy and promote scar tissue formation (Korntner et al., 2019). Therefore, some researchers regard neovascularization as one of the main pathological processes of tendinopathy and believe that the mechanical properties of the tendon are negatively correlated with the level of neovascularization.

PRP can provide not only angiogenic protein but also a variety of angiogenesis inhibitors, including endostatin, fibronectin, platelet factor 4 (PF4), thrombospondin-1 (TSP-1), α2-macroglobulin, plasminogen activator inhibitor-1, angiostatin, and TIMPs (Nurden et al., 2008). The activation of angiogenesis inhibitors acts as a negative feedback mechanism limiting the capillary number and the VEGF level within the tendon. For example, TSP-1 and endostatin can block VEGF signaling pathways and inhibit endothelial cell proliferation (Andia and Maffulli, 2013). PF4 has the strongest ability to inhibit angiogenesis among all chemokines, directly through the binding of CXCR3 receptors and indirectly through blocking VEGF and bFGF to their receptors (Andia et al., 2012). Therefore, PRP might regulate the capillary network in chronic tendinopathy. Finnoff et al. (2011) explored the efficacy of ultrasound-guided PRP injection in the treatment of chronic refractory tendinopathy. They reported that the tendon function improvement rate and the pain relief rate were respectively 68% and 58% in 41 patients (10 with upper-extremity tendinopathy and 31 with lower-extremity tendinopathy), and 84% of patients had an improvement in imaging echo texture (Finnoff et al., 2011). In addition, intratendon calcification was relieved in 64% of patients, and neovascularization was reduced in 82% of patients (Finnoff et al., 2011). For chronic Achilles tendinopathy, a random controlled clinical trial designed by Boesen et al. (2017) demonstrated that PRP in combination with 12-week eccentric exercise rehabilitation therapy was superior to eccentric exercise alone. Combination therapy has advantages in improving tendon function and reducing pain, thickness, and the number of capillaries within the tendon (Boesen et al., 2017). However, a random controlled clinical trial designed by de Vos et al. (2011) showed that PRP could not improve vascularization and the structure of the collagen bundle within diseased Achilles compared with the saline group.

More long-term, high-quality, multi-center, large-scale basic or clinical trials are needed to confirm the exact effect and specific mechanism of PRP in regulating the capillary network of chronic tendinopathy. Bevacizumab is a VEGF-targeted monoclonal antibody agent used to treat many metastatic cancers (Garcia et al., 2020). Basic studies have demonstrated that early injection of bevacizumab in collagenase-induced tendinopathy can modulate the capillary network of the affected area, reduce tendon thickness, and improve the consistency of collagen fiber alignment (Dallaudiere et al., 2013). On this basis, Dallaudiere et al. (2014) used bevacizumab in combination with PRP to treat tendinopathy and found that the tendon healing effect was better in the combination group than in the single PRP injection group. The authors declaimed that angiogenesis inhibitors can reduce neovascularization and secretion of MMPs and prostaglandins in the early stage of tendinopathy. In the later stage of tendon healing, PRP can provide rich active cytokines that will compensate for the reduced concentrations of growth factors due to the decreased angiogenesis, promote the recruitment of stem cells and fibroblasts, and boost collagen synthesis (Dallaudiere et al., 2014). Although angiogenesis is one of the key steps in the pathogenesis of tendinopathy, few studies have been conducted on inhibiting angiogenesis for tendinopathy treatment. Further studies are needed to determine whether it is necessary to develop a series of therapies aimed at inhibiting tendon angiogenesis.

### 3.4 Regulation of inflammation

The role of inflammation in the pathogenesis of tendinopathy has not been determined. In the past 20 years, tendinopathy has been described as a non-inflammatory degenerative disease. Similarly, the continuum model did not emphasize the specific role of inflammation in the pathological process of tendinopathy (Cook and Purdam, 2009; D'Addona et al., 2017). However, the development and application of modern cellular and molecular biotechnology have improved the understanding of the inflammatory process in tendinopathy. The relevant roles of various cytokines, immune cells, and non-immune cells (mainly tenocytes) in the inflammation of tendinopathy have been elaborated. This suggests that inflammation may play a key role in the progression of degeneration and may be a key target for the treatment of tendinopathy.

IL-1*β* is produced by macrophages or tenocytes under pathological conditions and is expressed in the early stage of tendon injury (Yang et al., 2005; Mobasheri and Shakibaei, 2013). IL-1*β* can induce the expression of inflammatory mediators such as cyclooxygenase (COX-2), prostaglandin E2 (PGE 2), and MMP-1 in tenocytes, which will promote matrix breakdown and negatively affect the mechanical properties of the tendon (Yang et al., 2005; Mobasheri and Shakibaei, 2013). IL-1*β* can also inhibit the tendonogenic differentiation and tendon-related gene expression (e.g., Scx, tenomodulin, and collagen) of TSC, counteracting the tendon healing process (Zhang et al., 2015). IL-6 is a multifunctional Th2 cytokine induced by IL-1*β* and TNF-*α* (Lin et al., 2006; Millar et al., 2010; Tang et al., 2018). It has the function of regulating tendon inflammation and the healing process. IL-6 and IL-1*β* mediate inflammatory response after tendon injury by activating the NF-*κ*B pathway (Abraham et al., 2019). Animal models showed that the NF-*κ*B pathway was closely related to tendinopathy (Abraham et al., 2019; Best et al., 2019). Knockdown of the key target NFKB1 can enhance the activity of NF-*κ*B and MAPK, which will promote the recruitment of macrophages, the proliferation of tenocytes, and the deposition of collagen at the repair site, thus enhancing the mechanical properties of the tendon (Abraham et al., 2019; Best et al., 2019). Moreover, IL-6 can promote the expression of COL1A1 in the tendon, indicating that IL-6 is involved in the regulation of tendon healing (Millar et al., 2010). Animal models of acute tendon injury showed that the TNF-*α* gene level increased gradually from 2 h to 9 days after injury and then decreased in roughly the second week (Morita et al., 2017). TNF-*α* can stimulate tenocytes to express a multitude of pro-inflammatory or anti-inflammatory mediators, such as IL-1*β*, TNF-*α*, IL-6, IL-10, and MMPs (John et al., 2010; Tang et al., 2018). TNF-*α* may even induce apoptosis of the tenocyte, which in turn inhibits the production of extracellular matrix (John et al., 2010; Tang et al., 2018). Han et al. (2017) designed an *in vitro* experiment that demonstrates that TNF-*α* has the function of inhibiting TSC proliferation and tendonogenic differentiation. However, the combination of TNF-*α* with TGF-*β*1 can promote proliferation and tendonogenic differentiation of TSCs. Moreover, IL-10, IL-4, IL-33, IL-18, TGF-*β*, VEGF, COX-2, PGE 2, and other cytokines have important regulatory effects on inflammatory progression and the tissue repair process in tendinopathy (Tang et al., 2018; Chisari et al., 2019; Arvind and Huang, 2021; Chisari et al., 2021) (Table 3).

**TABLE 3:** Cytokines implicated in tendinopathy.

Cytokine	Proposed function in tendinopathy
IL-1*β*	Pro-inflammatory cytokine induction, ECM remodeling, reduced type I collagen production, and inhibited tendonogenic differentiation of TSCs
IL-6	Increased total collagen synthesis and inflammation regulation
TNF-*α*	Increased pro-inflammatory cytokine production, reduced type I collagen production, and inhibited proliferation and tendonogenic differentiation of TSCs
IL-4	Promoted acute inflammatory processes and associated with ECM homeostasis
IL-18	ECM remodeling and immune cell recruitment
IL-33	ECM remodeling and increased type III collagen and cytokine production
TGF-*β*	Promoted collagen synthesis, neovascularization, and fibrosis/excessive scar formation; promoted immune tolerance; and decreased inflammation
VEGF	Induced inflammation, degraded the ECM, and promoted pathogenetic processes of degenerative tendon

(Hershey, 2003; Yang et al., 2005; Lin et al., 2006; Millar et al., 2009; John et al., 2010; Millar et al., 2010; Oliva et al., 2011; Mobasheri and Shakibaei, 2013; Campbell et al., 2014; Millar et al., 2015; Zhang et al., 2015; Han et al., 2017; Tang et al., 2018; Titan et al., 2019).

**FIGURE 4:**
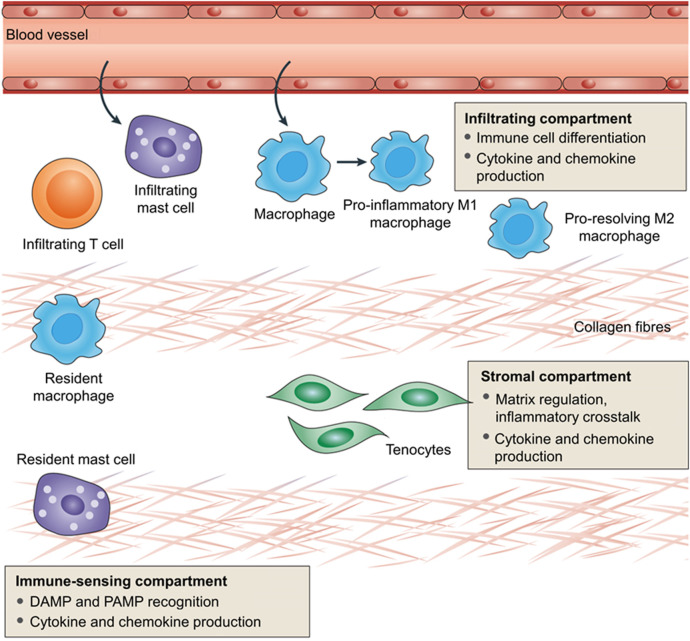
Immunobiology of tendinopathy. Inflammation in tendinopathy encompasses three distinct compartments involved in a complex network for tendon homeostasis. The infiltrating compartment includes influxing immune cells, probably recruited through stromal and resident immune cell activation. The infiltration of immune cells represents a homeostatic inflammation in normal circumstances but is aberrant in tendinopathic disease. The immune-sensing compartment comprises tendon-resident innate cells that act as sentinels to respond to initial tissue insult through damage-associated molecular patterns (DAMPs) and pathogen-associated molecular patterns (PAMPs). The centrally placed resident tenocytes sit in the influential stromal compartment, which is responsible for tissue remodeling and repair. Tenocytes can be driven toward an activated inflammatory phenotype and secrete cytokines and chemokines. These three compartments contribute to interactions between the inflammatory response and the extracellular matrix remodeling, which in turn comprise a balance between reparation and further degeneration within the tendon (Millar et al., 2017).

Millar et al. (2017) reviewed the role and status of immune cells (resident or infiltrated) and stromal fibroblasts in the inflammatory response in tendinopathy. The inflammatory sites of tendinopathy are creatively divided into three different cell compartments in this review: the stromal compartment, immune-sensing compartment, and infiltrating compartment (Millar et al., 2017). The infiltrating compartment accumulates a variety of circulating immune cells, including macrophages, NK cells, and lymphocytes. These circulating immune cells converge to the lesion by sensing pro-inflammatory cytokines released by resident immune cells and stromal fibroblasts. The immune-sensing compartment has resident immune cells, such as macrophages and mast cells. They can sense the damage/pathogen-related molecular patterns (DAMPs/PAMPs), take the lead in responding to tissue damage, and are activated by pro-inflammatory cytokines. Tenocytes located in the stroma are mainly responsible for tissue remodeling and repair. After sensing extracellular signals, tenocytes initiate downstream pathways and produce cytokines and chemokines in an autocrine or paracrine form, which can adjust tenocytes to an inflammatory phenotype (i.e., an “Activated” state). Consequently, extracellular matrix remodeling is accelerated. The interaction between different cell regions constitutes a complex environment of tendonitis, which affects the homeostasis of matrix degeneration and repair (Figure 4).

Among immune cells, the role of macrophages has been increasingly emphasized in the tendon inflammatory response, early tissue repair, and remodeling process (Lana et al., 2019). Traditionally, tendon macrophages have been classified into two phenotypes, M1 (classically activated macrophage) and M2 (alternatively activated macrophage), according to the surface receptors, functions, and cytokines expression (Mantovani et al., 2002). M1 macrophages promote tendon inflammation by producing cytokines, including CeC motif chemokine ligand 2, monocyte chemoattractant protein 1, inducible nitric oxide synthase, TNF-*α*, IL-12, IL-1*β*, and VEGF (Arnold et al., 2007; Ruffell et al., 2009; Ramachandran et al., 2012). M2 macrophages play an anti-inflammatory role, which can produce cytokines such as IL-1 receptor antagonist (IL-1RA), IL-4, IL-10, TGF-*β*, arginase 1, IGF-I, and PDGF (Arnold et al., 2007; Ruffell et al., 2009; Ramachandran et al., 2012). Therefore, M2 macrophages can inhibit tendon inflammation, inducing tenocytes to migrate to the injury site, and can regulate tendon repair and the fibrosis process (Mantovani et al., 2002). M1 macrophages reach the tendon within 24 h after injury and dominate in the subacute stage of tendinopathy (Dakin et al., 2012; Dakin et al., 2014). With the development of chronic tendon degeneration, the function of macrophages changes from pro- to anti-inflammation and tissue repairing (Dakin et al., 2012; Dakin et al., 2014). The number of M2 macrophages increases gradually (Dakin et al., 2012; Dakin et al., 2014). Murray et al. (2014) redefined macrophage types, emphasizing the role of key cytokines and signaling pathways. According to the new definition of macrophages, the interferon (IFN) and NF-*κ*B signaling pathways are activated in the early and middle stages of tendinopathy, whereas the signal transducer and activator of transcription 6 (STAT-6) and glucocorticoid receptor signaling pathways dominate in the later stage (Dakin et al., 2015). Furthermore, CD206 and ALOX15 mRNA pathways of the macrophage are significantly activated in tendinopathy patients with symptomatic relief after treatment compared with those still with symptoms (Dakin et al., 2015). This demonstrates the role of CD206 and ALOX15 pathways in relieving inflammatory pain in tendinopathy patients.

Tendon-resident stromal cells also play an important role in the initiation and maintenance of chronic inflammation. Tenocytes can be transformed from a quiescent state to an activated state by the inflammatory reaction of tendinopathy, being hypertrophic ones and highly expressing molecular markers such as toll-like receptor 4 (TLR4), interferon regulatory factor 1 (IRF1), IRF5, PDPN, CD106, and CD248 (Dakin et al., 2018). Meanwhile, activated tenocytes can synthesize and release cytokines, such as TNF-*α*, IL-1*β*, IL-6, IL-10, and VEGF (Serhan et al., 2008). Even when the inflammation of tendinopathy is reduced, the tenocytes remain in an inflammatory-sensitive state to some extent, which undoubtedly increases the risk of inflammation progression or recurrence (Dakin et al., 2018).

Platelets in PRP control the inflammatory response in tendinopathy by coordinating highly complex molecular signaling networks and cell–cell interactions (Andia and Abate, 2018). Platelets can store and release chemokines, including CXCL7 (NAP-2), PF4 (CXCL4), chemokine (C-C motif) ligand 5 (CCL-5), and CCL-2 (Flad and Brandt, 2010). These chemokines can regulate the biological behavior of a range of immune cells, including neutrophils, macrophages, and lymphocytes, maintaining an inflammatory microenvironment within the tendon. For example, CXCL7 has chemotaxis and activation effects on neutrophils (Ghasemzadeh et al., 2013). PF4 can switch macrophages from a pro-inflammation phenotype to a stromal synthesis phenotype, which in turn modulates inflammation in tendinopathy and repair response (Gleissner et al., 2010). CCL-2 and CCL-5 can promote the migration of monocytes and T cells across capillaries by regulating the activity of CCR2, CCR1, and CCR5 (Stalman et al., 2015). Conversely, Zhang et al. (2013) found that HGF in PRP could antagonize the pro-inflammatory effect of IL-1*β*; inhibit the expression of COX-1, COX-2, and PGE 2 synthase in tenocytes; and reduce the production of PGE 2. Meanwhile, HGF can inhibit the inflammatory reaction by increasing the expression of I*κ*B*α* (Bendinelli et al., 2010). I*κ*B*α* is an inhibitor of NF*κ*B; the latter is an inflammatory modulator involved in tendinopathy and is upregulated within tenocytes at the site of tendon inflammation (de Oliveira et al., 2013). Moreover, IGF-1 and PDGF in PRP can inhibit the production of I*κ*B kinase to prevent intratendon inflammation (Zhang and Wang, 2014). TGF-*β*, an anti-inflammatory cytokine in PRP, has been proven to control local inflammation (Jo et al., 2012). Given that platelets contain both pro- and anti-inflammatory cytokines, LP-PRP might have a complex regulatory mechanism in the inflammatory response of tendinopathy.

Recent data suggest that the microenvironment within the tendon is crucial for the ultimate effect of LP-PRP in regulating inflammation. An *in vitro* experiment designed by Jo et al. (2018) demonstrated that PRP could only exert an anti-inflammatory effect on tenocytes pretreated with IL-1*β* or derived from degenerative tendons. Conversely, PRP can promote an inflammatory response (Jo et al., 2018). In other words, in the normal, non-inflammatory microenvironment, PRP can upregulate the expression of IL-1*β*, Cox-2, membrane-bound prostaglandin E2 synthase 1 (mPGES-1), and downstream MMPs; downregulate anti-inflammatory cytokine levels; and thus induce inflammation and pain. However, in the inflammatory microenvironment, PRP can downregulate the expression of pro-inflammatory cytokines and MMPs, upregulate anti-inflammatory cytokine levels, and promote matrix synthesis, thus inhibiting the inflammatory response and enhancing the repair response. This finding could help explain some of the conflicting claims in previous studies about the anti-inflammatory effect of LP-PRP (El-Sharkawy et al., 2007; Zhang et al., 2013; Andia et al., 2015; Zhang L. et al., 2016; Hudgens et al., 2016). For example, Hudgens et al. (2016) reported that for normal tendon fibroblasts, LP-PRP treatment downregulated the expression of EGR1, EGR2, tenomodulin, and Scx, which are closely correlated to extracellular matrix formation. Furthermore, LP-PRP treatment activated TNF-*α* and NF*κ*B signaling pathways, inducing an inflammatory response in tendon fibroblasts (Hudgens et al., 2016). LP-PRP also promoted the expression of autophagy-related genes and reactive oxygen species (ROS) genes and activated the oxidative stress pathways (Hudgens et al., 2016). However, Andia et al. (2015) found that for the inflammatory-activated tenocytes induced by IL-1β, PRP treatment downregulated the expression of IL-6/CXCL6, IL-6R, and IL-8/CXCL8 and decreased the secretion of immunomodulatory proteins IL-8/CXCL8, IL-6/CXCL6, and MCP-1/CCL2. Likewise, Li et al. (2020) reported that the injection of LP-PRP into the degenerative Achilles of rabbits could significantly reduce the release of pro-inflammatory cytokine IL-6 and decrease the expression of MMP-1 and MMP-3. In addition, LP-PRP increases the expression of TIMP-1 and maintains the balance of MMPs and TIMPs in the tendon, which is beneficial to the maturation of the extracellular matrix (Li et al., 2020).

In general, M1 macrophages can be stimulated by LPS and IFN-*γ*, whereas M2 macrophages can be stimulated by Th2 lymphocytes IL-4 and IL-10 (Wynn and Vannella, 2016). Therefore, macrophage activity and functional phenotype might be directly affected by PRP components and their concentration levels, especially leukocytes. The biological basis for this view is that LR-PRP and LP-PRP have different concentrations of pro- and anti-inflammatory mediators. LR-PRP contains higher concentrations of pro-inflammatory mediators, such as TNF-*α*, IL-6, and IFN-*γ* (Sundman et al., 2011; Braun et al., 2014). However, LP-PRP can increase the concentrations of anti-inflammatory mediators such as IL-4 and IL-10 in the local tissue (Sundman et al., 2011; Braun et al., 2014). For example, a random controlled trial by Nishio et al. (2020) found that in the injured patellar tendon model, the number of M1 macrophages increased significantly on days 4 and 7 after treatment with LR-PRP or LP-PRP compared with the control group. However, the M1 macrophage count/M2 macrophage count decreased significantly on days 7 and 14 after surgery only in the LP-PRP group (Nishio et al., 2020). This proves that LP-PRP has the function of anti-inflammation and acceleration of the recovery of the degenerative tendon. In summary, LR-PRP might elicit a more severe inflammatory response when considered in terms of macrophage alone. Rapid infiltration of inflammatory cells might stimulate the tissue repair process more rapidly. On the contrary, LP-PRP has a strong potential to induce anabolism, which is beneficial in enhancing tenocyte proliferation and promoting tendon remodeling.

The presence and concentration of leukocytes (mainly neutrophils) in PRP must be considered in the treatment of tendinopathy. The leukocyte number in LP-PRP is reduced by 22-fold compared with baseline, almost eliminating this cellular component (Fitzpatrick et al., 2017b). However, LR-PRP can enrich leukocytes 3–5 times the baseline level (Fitzpatrick et al., 2017b). In addition, PRP produced by different preparation systems has different proportions of neutrophils, lymphocytes, and monocytes (Fitzpatrick et al., 2017b). The role of leukocytes is controversial. Some researchers claim that leukocytes in PRP have some positive and antibacterial effects. They can regulate the local immune microenvironment, which is helpful to prevent or control the infection of the injured tendon (Castillo et al., 2011). Leukocytes can also increase the concentration of growth factors in PRP by directly releasing or indirectly stimulating platelets to release growth factors (Zimmermann et al., 2001). In addition, leukocytes are able to promote angiogenesis and recruit macrophages at the lesion site to transform chronic low-grade inflammation in tendinopathy into regenerative inflammation (Millar et al., 2017; Chisari et al., 2021). However, other researchers believe that leukocyte is not an ideal component of PRP. Leukocytes can not only release multiple MMPs, but also stimulate tenocytes and TSCs to express MMP-1, MMP-3, and MMP-13, which has a negative effect on tendon matrix deposition (McCarrel et al., 2012; Cross et al., 2015; Zhou et al., 2015; Zhang L. et al., 2016; Bonilla-Gutierrez et al., 2018). Meanwhile, leukocyte infiltration can increase the levels of IL-1*β*, IL-6, TNF-*α*, and IL-8 in the tendon. It might inactivate growth factors, such as PDGF and TGF-*β*1, further worsen the microenvironment of the degenerative tendon, and aggravate the inflammatory reaction (McCarrel et al., 2012; Cross et al., 2015; Zhou et al., 2015; Zhang L. et al., 2016; Bonilla-Gutierrez et al., 2018). In addition, macrophages and other inflammatory cells can release a variety of COX, such as COX-1 and COX-2. Under the influence of high concentrations of COX-1 and COX-2, a large amount of PGE2 is produced and enriched in the affected site by mPGES, resulting in vasodilatation and pain (Ferreira et al., 1978; Williams, 1979; Smith, 1989).

**FIGURE 5:**
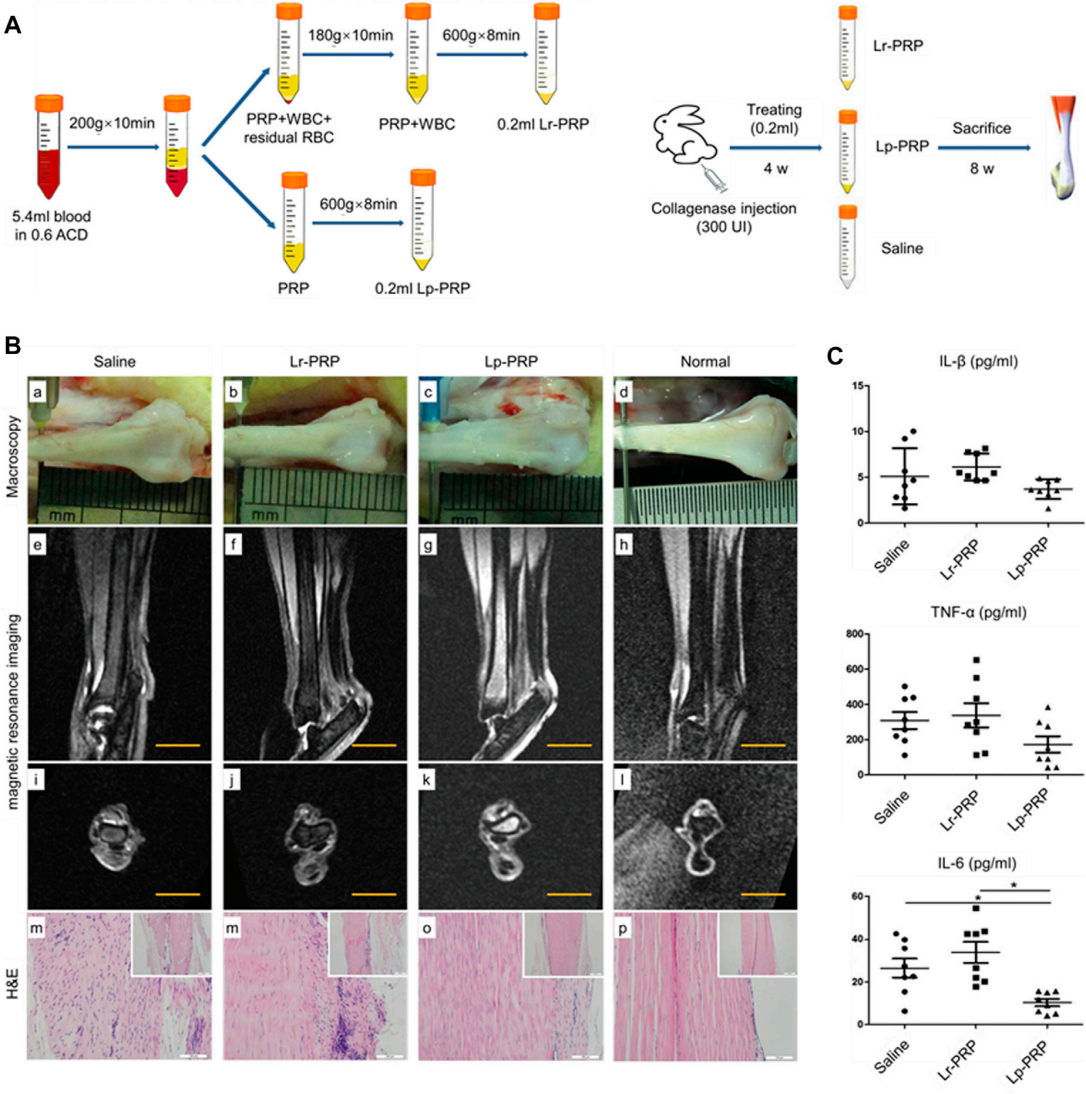
Anti-inflammatory effect of the intratendon delivery of leukocyte-poor PRP (LP-PRP) and leukocyte-rich PRP (LR-PRP) in a rabbit Achilles chronic tendinopathy model *in vivo*. **(A)** Flowchart of the preparation process of two kinds of PRP and the study design. **(B)** Gross view of the tendon in the saline, Lr-PRP, Lp-PRP, and normal groups (a–d). The Achilles tendons of the Lr-PRP group appeared to be pallid and displayed more swelling compared with the Lp-PRP group. Sagittal and transverse magnetic resonance imaging (MRI) T2-weighted images of the Achilles tendon in the saline, Lr-PRP, Lp-PRP, and normal groups (e–l). MRI revealed that the Lr-PRP and saline groups displayed higher signal intensities compared with the Lp-PRP group with T2 mapping, representing inflammatory edema at the lesion site. **(C)** Detection of IL-1b, TNF-a, and IL-6 by enzyme-linked immunosorbent assay, indicating Lr-PRP elicited more catabolic cytokine release than Lp-PRP at the local lesion site of the tendon (Yan et al., 2017).

Recent studies suggest that whether leukocytes in PRP are beneficial to tendon healing depends on the severity of tendinopathy. LR-PRP can further improve the therapeutic effect on tendinopathy in the early stage, whereas LP-PRP is more effective in the middle and late stages of tendinopathy. Yan et al. (2017) established a rabbit model of Achilles tendinopathy by injection of collagenase (Figure 5). In the 4th week after collagenase injection, LP-PRP, LR-PRP, and saline were utilized respectively to treat the degenerative Achilles (Yan et al., 2017). Yan et al. (2017) found that the LP-PRP group had higher histological scores, better structure and alignment of collagen fibers, and fewer pro-catabolic inflammatory factors than the other two groups. On this basis, Jiang et al. (2020) applied LP-PRP, LR-PRP, and saline, respectively, to treat the degenerative Achilles in rabbits in the 1st week after collagenase injection. They found that the Achilles treated with LR-PRP had more mature collagen fibers and better mechanical properties. Similarly, Li et al. (2020) treated tendinopathy with LR-PRP and saline separately in the 1st and 4th weeks after collagenase injection. The effect was evaluated by cytokine quantification, MRI, histological analysis, and transmission electron microscopy in the 6th week after collagenase injection (Li et al., 2020). The results show that LR-PRP can achieve the best therapeutic effect in the early stage of tendinopathy. The LR-PRP group in the 1st week had the smallest lesion area, the lowest MRI signal intensity, the largest mean diameter of collagen fibers, the highest expression level of type I and III collagen genes, and the best histological score (Li et al., 2020). However, the injection of LR-PRP in the late stage of tendinopathy can induce excessive catabolism and inflammation, which is not beneficial for the repair of tendinopathy (Li et al., 2020).

## 4 The use of PRP in clinical practice or clinical trials

Over the past few years, many studies have been conducted to evaluate the effectiveness of PRP in treating musculoskeletal conditions. A large number of basic research and clinical trials have proven the curative efficacy and safety of PRP for tendinopathy, although some of the results show that PRP is not so effective (de Vos et al., 2010b; Kearney et al., 2021; Dai et al., 2023). Consequently, there is controversy about the benefits of PRP for patients with tendinopathy. Currently, according to the mainstream view, such controversy is attributed to a lack of unified standards for the preparation process and the way of activation and administration of PRP in the research, as well as the pathological process of tendinopathy. Therefore, some researchers suggest recording the characterization details in clinical trials. These characterization details include the preparation methods (parameters of centrifugation and activation status/activator used); composition (concentration of platelets, leukocytes, and relative growth factors); and dosing methods (volume of therapeutic dose and number of doses administered) of PRP (Fitzpatrick et al., 2017a; Chahla et al., 2017; Murray et al., 2017; Rodeo, 2019). Meanwhile, researchers should identify the onset site and properly evaluate the current pathological process (early reactive tendinopathy, tendon disrepair, or degenerative tendinopathy) (Nourissat et al., 2015b; Filardo et al., 2018). This section discusses and analyzes the topics above, with the aim of establishing norms in clinical studies to allow comparison among studies and provide reproducibility.

The difference in the composition of PRP may be one of the main reasons for the controversy. The final ingredients of PRP are affected by two interrelated factors. The first is the concentration of platelets, leukocytes, and cytokines in the whole blood of patients (Mazzocca et al., 2012). The second is the anticoagulation methods, speed, rounds, and duration of the centrifugation process for different PRP preparation systems (DeLong et al., 2012; Chahla et al., 2017). For example, significant differences exist in the platelet capture efficiency; leukocyte concentration; and PDGF-AB, PDGF-BB, and VEGF concentrations in the PRP prepared by three different systems (Cascade, Magellan, and Biomet GPS III) (Castillo et al., 2011). However, there is a general lack of detailed recording of PRP preparation schemes and composition in the current clinical trials, making it impossible to reproduce the trial or compare the efficiency of PRP prepared by different systems (Abate et al., 2012). A systematic review of PRP application in orthopedic practice shows that among the 105 clinical studies included, only 11 (10%) submitted comprehensive PRP preparation reports, and 17 (16%) reported PRP composition (Chahla et al., 2017). Another recent study found that only a third of studies among all specialties, including musculoskeletal specialties, provided details on the PRP processing and characteristics, with only half of those studies performing leukocyte analysis (Nazaroff et al., 2021). Classification systems that can evaluate PRP are available now. The platelet, activation, white blood cell (PAW) classification system designed by DeLong et al. (2012) is regarded as a feasible PRP classification system. The PAW system evaluates the platelet count, activation methods, and leukocyte and neutrophil concentrations of PRP. Therefore, it can accurately compare the efficacies of PRP with different compositions and facilitate further exploration of optimal PRP ingredients. The concentration level for some other important growth factors, such as PDGF, TGF-*β*, and VEGF, should also be considered (Chen et al., 2022).

PRP can maintain an anticoagulation state for up to 8 h. However, PRP must be activated to release platelet granule content to exert treatment effects. The activated RPR will form a growth factor-rich blood clot that can release high-concentration growth factors and play a positive role in promoting the proliferation and migration of tenocytes and the formation of a tendon matrix. The choice of PRP activators and the activation duration will affect the release rate of growth factors, causing differences in the overall efficacy (Chen et al., 2018). Bovine thrombin and calcium chloride are the two most widely used PRP activators. Bovine thrombin is not recommended because it causes faster release of the growth factors from the platelet and has toxicity on human bodies. Marx (2004) showed that bovine thrombin caused 70% of the growth factors from platelet to release in 10 min and nearly 100% of the growth factors to release in 1 h. Furthermore, the use of bovine thrombin as the clotting initiator could put patients at risk of coagulopathy (Alsousou et al., 2013). Calcium chloride is safe but with a longer activation time (at least 20 min). Preparing and using calcium chloride solution to activate PRP will not expose patients to viruses or prions. The addition of calcium chloride results in the formation of a dense fibrin matrix, and intact platelets are subsequently trapped within (Abate et al., 2012). This system minimizes platelet activation, and the result is a slow release of growth factors over a 7-day period. Endogenous type I collage is also considered to be able to activate platelets within PRP to release growth factors (DeLong et al., 2012). An *in vitro* experiment compared the differences in activation effects on human PRP between type I collagen and bovine thrombin (Fufa et al., 2008; DeLong et al., 2012). This experiment found that from day 1 to day 10, the release volumes of PDGF-AB and VEGF for the two groups were approximately equal. However, from day 1 to day 5, the release volume of TGF-*β* for the type I collagen group was higher, and the shrinkage of the blood clot was significantly inhibited. This proved that type I collagen is effective in activating PRP. Photoactivation is considered an alternative to platelet activation. A recent study has compared the light-activated PRP with the chloride-activated PRP (Irmak et al., 2020). Studies have shown that the activation of platelets using a polychromatic light source (wavelengths in the range of 600–1,200 nm, near-infrared region) for 10 min will cause a sustainable release of growth factors for 28 days. Compared with the calcium-chloride-activated PRP, the light-activated PRP maximizes the release time of growth factors, such as PDGF, FGF, and TGF-*β*1, with a higher release volume.

During the treatment of tendinopathy, how to concentrate PRP at the lesion site efficiently and safely is one of the key factors that affect treatment outcomes. PRP injection to the osteotendinous junction, tendon, or myotendinous junction, respectively, will produce diverse outcomes, indicating the efficacy varies by administration positions (Anitua et al., 2009). Ultrasound-guided musculoskeletal procedures have become an effective administration therapy and are more widely used for the treatment of musculoskeletal diseases (Barile et al., 2016). Ultrasound-guided PRP injection technique allows the surgeons to control the depth of needle insertion and accurately deliver PRP to the lesion site of tendons (de Vos et al., 2010a). Some researchers use a peppering injection technique to eliminate the range of inflammation and stimulate the self-healing process of the tendon (Edwards and Calandruccio, 2003). In recent years, various PRP delivery systems have been successfully developed, including injectable biodegradable gelatin hydrogel microspheres and biological macromolecule hybrid hydrogels (Nagae et al., 2007; Saito et al., 2009; Lu et al., 2019). Choi et al. (2020) used microfluidics to fabricate biodegradable PRP-loaded polyethylene glycol microspheres. This system dramatically slowed the degradation of polyethylene glycol, achieved the sustained release of growth factors within PRP, and to some extent, resolved the fast-release problem of growth factors with a single injection.

The number of PRP administrations is also a key factor. Research shows that a single injection and multiple injections bring different outcomes in the treatment of tendinopathy (Zayni et al., 2015). A meta-analysis discussing the efficacy of PRP on patellar tendinopathy has shown that a single injection of PRP delivers better outcomes in the short term, whereas multiple PRP injections demonstrate more obvious benefits in the long term (Andriolo et al., 2019). Therefore, personalized PRP administration protocols should be given to patients based on their tolerance and the severity of symptoms. For chronic tendinopathy with severe symptoms, it is suggested to combine multiple PRP injections with rehabilitation training (Kon et al., 2009).

PRP application may offer different outcomes according to the tendon disorders considered. The specific anatomical areas of the tendon and tendinopathy pathological progress could be potential factors that affect the efficacy of PRP. Patellar tendons seem to benefit from PRP injections. A systematic review, including 70 studies with 2,350 patients, has shown that multiple PRP injections are an appropriate option for patients with chronic patellar tendinopathy (Andriolo et al., 2019). Dragoo et al. (2014) compared clinical outcomes in patellar tendinopathy after a single ultrasound-guided LR-PRP injection *versus* dry needling. At 12 weeks after treatment, the PRP group showed a better Victorian Institute of Sports Assessment score for patellar tendinopathy than the dry needling group. No significant difference was observed in the scores of the two groups at 26 weeks. This indicates that patellar tendinopathy can benefit from PRP in the early stage of the treatment. However, results in the Achilles tendon do not justify the application of the evaluated platelet concentrates, neither conservatively nor surgically. A meta-analysis, including five randomized control trials with 189 patients, has reported that PRP injection therapy for chronic Achilles tendinopathy is not better than placebo treatment (Liu et al., 2019). The first double-blind, randomized control trial was authored by the group of de Vos et al., who compared a single non-activated intratendinous injection of PRP in patients with chronic midportion Achilles tendinopathy against a single injection of saline (de Vos et al., 2010b; de Jonge et al., 2011). Although an overall improvement was described in both groups, the authors failed to show any significant inter-group differences. Similar results were also reported by Krogh et al. (2016), who found no difference between PRP and saline solution 3 months after the injective treatment. Lateral elbow tendinopathy showed improvement with the application of PRP in most of the high-level studies. A meta-analysis, including seven randomized control trials with 515 patients, showed that 6 months after treatment, the PRP group was better than the local corticosteroid injection group in relieving pain and improving elbow function (Xu et al., 2019). Another systematic review, including nine meta-analyses with 8,656 patients, revealed that corticosteroid injection can improve the function and relieve the pain for patients with lateral epicondylitis in the short term, and PRP injection is the most effective therapy in the middle term (Houck et al., 2019). The findings on the PRP conservative treatment of rotator cuff are still too limited to provide viable indications. A systematic review, including five randomized control trials, showed that for patients with chronic rotator cuff injury, PRP injections may not be beneficial in the short term. When directly compared with exercise therapy, PRP does not result in superior functional outcomes, pain scores, or range of motion (Hurley et al., 2019). With respect to the surgical application, PRP has been widely used as a biological enhancer during or immediately after arthroscopic rotator cuff repair. There is more consistent literature with an overall agreement on the benefit of PRP surgical augmentation (Jo et al., 2013; Malavolta et al., 2014; Pandey et al., 2016).

From a clinical viewpoint, an important consideration is the considerable pathogenetic heterogeneity of tendinopathy. The pathological characteristics of treated tendinopathy are not always described in detail in the published work, which limits the validity of study conclusions and hinders comparisons across studies (Nourissat et al., 2015b). Distinct injury–repair reactions will occur in different pathological stages of tendinopathy. An appropriate description of the patient’s medical history and an accurate evaluation of the tendinopathy pathological stage based on the continuum model is necessary in the manuscripts. Useful information could no doubt be obtained by performing studies in narrower indications, probably at an early stage of the tendon disease. Currently, the available scientific evidence does not warrant the use of PRP for the first-line treatment of tendinopathy. PRP therapy may deserve consideration in specific tendinopathy subtypes after the failure of ultrasound-guided corticosteroid injections. Nevertheless, further studies are needed to define these potential indications and the optimal treatment protocols.

Moreover, considering tendinopathy in competitive athletes and the possible use of PRP for tissue recovery, the world anti-doping agency (WADA) position on this treatment is particularly important. According to the 2023 list of prohibited substances and methods, growth factors and growth factor modulators affecting muscle, tendon, or ligament protein synthesis/degradation, vascularization, energy utilization, and regenerative capacity of fiber type switching are prohibited at all times (in- and out-of-competition). Activated PRP contains a variety of growth factors, including PDGF, HGF, VEGF, and FGF, which should be prohibited in principle. For competitive athletes with tendinopathy, using PRP for treatment is required to be in compliance with the appropriate international standards for therapeutic use exemptions and to obtain the legal right for PRP therapy. Notably, the latest international standard for therapeutic use exemption stipulates that athletes may be granted a therapeutic use exemption if (and only if) they can show on the balance of probabilities that each of the following conditions is met. a) The prohibited substance or method in question is needed to treat a diagnosed medical condition supported by relevant clinical evidence. b) The therapeutic use of the prohibited substance or prohibited method will not, on the balance of probabilities, produce any additional enhancement of performance beyond what might be anticipated by a return to the athlete’s normal state of health following the treatment of the medical condition. c) The prohibited substance or method is an indicated treatment for the medical condition, and there is no reasonably permitted therapeutic alternative. d) The necessity for the use of the prohibited substance or method is not a consequence, wholly or in part, of the prior use of a substance or method which was prohibited at the time of such use. Competitive athletes with tendinopathy and their coaches should decide whether to use PRP for tendinopathy after giving full consideration to the regulations above and are required to submit a request to the competent authority to obtain the legal right to apply PRP therapy.

## 5 Conclusion

PRP is self-derived and easily accessible without risk of immune rejection or disease transmission. PRP has been gradually applied in the treatment of many diseases, especially in the field of musculoskeletal, and has shown certain curative effects. Researchers have conducted several trials over the past decade to assess the efficacy of PRP for tendinopathy. However, some experimental results are conflicting, and there is no consensus regarding the efficacy of PRP for tendinopathy. The controversy over the results can be attributed to differences among studies, known as “4D,” which means differences in PRP ingredient, mode of administration, donor (patient), and disease (type, stage). This article focuses on the pathogenesis of tendinopathy and attempts to combine the PRP mechanism with the pathological process of tendinopathy, thereby better understanding how PRP treats tendinopathy and the reasons for the differences in the clinical outcomes. Moreover, based on fundamental research, this article integrates the specific molecular and cellular mechanisms by which PRP manipulates tendon healing, including promoting tendon regeneration and modulating inflammation in tendinopathy. Future studies should further elucidate PRP and tendinopathy/tendon regeneration interaction, as well as the optimal PRP component and application mode, depending on the type and stage of tendinopathy. Finally, although the safety of this biological approach obtains overall support, reports about adverse consequences still exist. Therefore, the prognosis of PRP in all studies needs to be documented to clearly identify possible contraindications. Based on current literature, PRP should not be used indiscriminately in clinical practice for tendinopathy but rather as a second-line treatment until further evidence provides a clear indication. Clinicians should be aware of the different therapeutic potentials of PRP for different types or stages of tendinopathy and should provide suitable treatment for patients with tendinopathy.
